# Simultaneous Activation of Erk1/2 and Akt Signaling is Critical for Formononetin-Induced Promotion of Endothelial Function

**DOI:** 10.3389/fphar.2020.608518

**Published:** 2021-01-11

**Authors:** Jinjun Wu, Muyan Kong, Yanmei Lou, Leyan Li, Chunlin Yang, Huifang Xu, Yuqi Cui, Hong Hao, Zhenguo Liu

**Affiliations:** ^1^ Joint Laboratory for Translational Cancer Research of Chinese Medicine of the Ministry of Education of the People’s Republic of China, International Institute for Translational Chinese Medicine, Guangzhou University of Chinese Medicine, Guangzhou, China; ^2^ Center for Precision Medicine and Division of Cardiovascular Medicine, University of Missouri School of Medicine, Columbia, MO, United States

**Keywords:** formononetin, angiogenesis, ERK1/2, Akt, eNOS, NO

## Abstract

Formononetin (FMNT) is a major bioactive compound from *Astragalus membranaceus* (Fisch.) Bunge, and has been widely used to treat conditions related to vascular insufficiency. However, the molecular mechanism for the therapeutic effect has not been well defined. This study aimed to investigate the effect and mechanism of FMNT on endothelial function. The potential targets and signaling pathways of FMNT in the setting of ischemia were predicted using network pharmacology analysis. Human umbilical vein endothelial cells (HUVECs) were used for the *in vitro* studies and C57BL/6 mice were used for *in vivo* experiments. The results of the network pharmacology analysis showed that multiple signaling molecules including MAPK and PI3K-Akt pathways could be involved in the pharmacological actions of FMNT against ischemic diseases. The experimental validation data showed that FMNT significantly promoted the growth, proliferation, migration and tube formation of HUVECs in association with activation of endothelial nitric oxide synthase (eNOS) and promotion of intracellular nitric oxide (NO) production. FMNT also markedly activated Erk1/2 and Akt signaling in HUVECs. The enhanced endothelial function by FMNT was abolished when the cells were pre-treated with eNOS inhibitor. FMNT-induced eNOS/NO activation, endothelial function and angiogenesis was also effectively attenuated when Erk1/2 or Akt signaling pathway was inhibited. In addition, FMNT significantly promoted wound healing in C57BL/6 mice associated with activation of Erk1/2 and Akt signaling. Enhanced wound healing by FMNT in mice was prevented when eNOS-, Erk1/2, or Akt-medicated signaling was inhibited. Moreover, when Akt signaling was inhibited in HUVECs, FMNT was still able to activate Erk1/2 signaling without promotion of endothelial function. Similarly, FMNT could activate Akt signaling with no change in endothelial function when Erk1/2 signaling was attenuated in HUVECs. Conclusively, the present study demonstrated that FMNT significantly enhanced endothelial function and promoted angiogenesis *in vitro* and *in vivo* through activating Erk1/2- and Akt-mediated eNOS/NO signaling pathway. The data also suggested that simultaneous activation of Erk1/2 and Akt signaling was required for FMNT-induced promotion of endothelial function. Results from the present study might provide support and evidence for the application of FMNT during the clinical treatment of conditions related to vascular insufficiency.

## Introduction

Endothelial cells play a vital role in the regulation of vascular function including angiogenesis (Carmeliet, 2003; Bauer et al., 2005; Falanga, 2005). Many flavonoids from herbal medicine have been shown to promote angiogenesis (Cho et al., 2008; Tang et al., 2010; Li et al., 2011). Formononetin (FMNT) is one of the major isoflavonoids from *Astragalus membranaceus* (Fisch.) Bunge, a Chinese herbal medicine that has been widely used for over 2000 years for treating a variety of conditions including anemia, fever, chronic fatigue, allergies, loss of appetite, uterine bleeding, uterine prolapse, ischemic diseases, and wound healing (Fu et al., 2014; Wu et al., 2018; Lou et al., 2019). Experimental and clinical studies have shown that FMNT enhances early fracture healing through stimulating angiogenesis by up-regulating VEGFR-2/Flk-1 in rat, and promotes endothelial function and wound healing in association with increased production of growth factors and activation of estrogen receptor alpha-enhanced ROCK pathway (Huh et al., 2009; Huh et al., 2011; Li et al., 2015). However, the molecular mechanism(s) for FMNT-induced angiogenesis has yet to be fully elucidated.

Nitric oxide (NO) is essential for endothelial function including cell growth, migration, and angiogenesis (Gooch et al., 1997; Murohara et al., 1998; Parenti et al., 1998; Dimmeler et al., 2000; Morbidelli et al., 2003; Naseem, 2005). Endothelial nitric oxide synthase (eNOS) is involved in NO production in endothelial cells (Dimmeler et al., 2000). Several kinases including protein kinase B (Akt) and extracellular regulated protein kinase 1/2 (Erk1/2) enhance eNOS phosphorylation (Ser1177) (Wang et al., 2013; Ahmad et al., 2018; Lee et al., 2018). Activation of phosphatidylinositol 3-kinase/protein kinase B (PI3K/Akt) signaling pathway results in sustained NO production through eNOS phosphorylation, promotes endothelial function including vasorelexation, migration, and angiogenesis (Kawasaki et al., 2003; Kaufeld et al., 2014; Anwar et al., 2017). Erk1/2 signaling is also critical to the regulation of eNOS phosphorylation and NO production, and endothelial function including survival, proliferation, migration, angiogenesis, and tube formation (Buckley et al., 1999; Meadows et al., 2001; Wang et al., 2013; Zhang et al., 2017). However, it is unknown whether FMNT could enhance endothelial function via PI3K/Akt- and/or Erk1/2-mediated eNOS/NO signaling pathway.

In the present study, *in vitro* and *in vivo* experiments were designed to investigate the role of Akt and Erk1/2 signaling in mediating the effect of FMNT on endothelial function. The objectives were: 1). to predict the potential targets and signaling pathways of FMNT in the setting of ischemia using network pharmacology analysis; 2). to evaluate the effects of FMNT on endothelial function including proliferation, migration, and tube formation, and eNOS phosphorylation and NO production in endothelial cells; 3). to investigate the role of PI3K/Akt and Erk1/2 in mediating the effect of FMNT on eNOS/NO signaling and endothelial function; and 4). to evaluate the effect of FMNT on wound healing. Human umbilical vein endothelial cells (HUVECs) were used for *in vitro* studies, and C57BL/6 mice were used for *in vivo* experiments.

## Materials and Methods

### Chemicals and Reagents

FMNT (98% purity) was purchased from Frontier Scientific, Inc. (Newark, DE, USA). Recombinant human VEGF-165 (#293-VE-010/CF) was from R&D Systems (Minneapolis, MN, USA). Endothelial cell medium kit was purchased from Sciencell Research Laboratories (Carlsbad, CA). Cell Counting kit 8 (CCK8) was obtained from Dojindo Laboratories (Kumamoto, Japan). Click-iT™ EdU Alexa Fluor™ 488 Imaging Kit, goat anti-Rabbit IgG (H + L) Cross-Adsorbed Secondary Antibody Alexa Fluor^®^ 488 conjugate, Alexa Fluor™ 555 Phalloidin, DAPI (4',6-Diamidino-2-Phenylindole, Dihydrochloride), DAF-FM Diacetate (4-Amino-5-Methylamino-2',7'-Difluorofluorescein Diacetate), and Lipofectamine™ 3000 Transfection Kit were from Invitrogen (Carlsbad, CA, USA). Cytoselect 24-well cell migration kit was obtained from Cell Biolabs, lnc. (San Diego, CA, USA). Matrigel Basement Membrane Matrix (356234) was from BD Bioscience (San Jose, CA, USA). L-nitro-arginine methyl ester (L-NAME) were from MedChem Express (Monmouth Junction, NJ, USA). PD98059 (#9900), LY294002 (#9901), Phospho-eNOS (Ser1177, #9571), eNOS (#32027), p-Akt (Ser473, #4060), Akt (#9272), p-Erk1/2 (Thr202/Tyr204, #9101), Erk1/2 (#9102), GAPDH (#5174), control siRNA (siCon, #6568), Erk1/2-specific siRNA (siErk1/2, #6560), and Akt-specific siRNA (siAkt, #6211) were from Cell Signaling Technology Inc. (Beverly, MA). All other chemicals were of analytical reagent grade or better.

### Cell Culture

HUVECs were purchased from Sigma-Aldrich (St. Louis, MO). The cells were cultured in endothelial cell medium (ECM) supplemented with 5% (v/v) fetal bovine serum (FBS), 1% (v/v) endothelial cell growth supplement (ECGS) and 1% (v/v) penicillin/streptomycin solution (P/S) (ScienCell Research Laboratories, Inc., Cat No. 1001), at 37°C in a humidified atmosphere with 5% CO_2_. The medium was changed every other day unless specified.

### Animals and Treatments

All animal experiments were performed in accordance with the “Guide for the Care and Use of Laboratory Animals of the US National Institutes of Health”. Animal experiment protocols were reviewed and approved by Guangzhou University of Chinese Medicine Animal Care and Use Committee (Guangzhou, China) (IITCM-20190306). Male specific pathogen-free (SPF) C57BL/6 mice (4–6 weeks old, 18–22 g) were from the Laboratory Animal Center of Sun Yat-Sen University, Guangzhou, China. Mice were kept in the SPF animal facility (License number: SYXK (GZ) 2019-0144) at the International Institute for Translational Chinese Medicine, Guangzhou University of Chinese Medicine (Guangzhou, China). Mice were randomly divided into eight groups to investigate the effect of FMNT on wound healing. After general anesthesia with isofluane, a full-thickness excisional wound (10 mm) was created on the dorsal back of each mouse using a biopsy punch (a scissors) after hair removal and sterilization. Immediately after wound creation surgery, FMNT (12.5, 25 and 50 μΜ), VEGF (50 ng/ml), vehicle (PBS), FMNT (50 μΜ) plus L-NAME (200 μM), FMNT (50 μΜ) plus LY294002 (20 μM) or FMNT (50 μΜ) plus PD98059 (40 μM) was injected into five different sites of intact dermis next to the wound once a day (the total volume of five injections per wound was 50 μl) for 10 consecutive days. Injection sites were about 1 cm away from the wound edge to avoid leakage. The wound areas were measured immediately and at postoperative day 2, 4, 6, 8, and 10. Wound area reduction was calculated as the percentage of the original wounded area. On day 10, mice were euthanized to collect tissue samples at the site of healed skin of each mouse for further analysis.

### CCK8 Assay

Cell growth was assessed using CCK8 assay following manufacturer’s instructions. In brief, HUVECs were seeded at a density of 5 × 10^3^ per well in 96-well plates and cultured overnight. The cells were incubated with the vehicle, or FMNT (2.5–100 μM) for 24 h or pretreated with L-NAME (100 μM), LY294002 (20 μM), or PD98059 (10 μM) for 1 h before incubation with FMNT (20 μM) for 24 h with VEGF (40 ng/ml) as a positive control. At the end of incubation, CCK8 reagents were added to each well and incubated for 3 h at 37°C. The spectrophotometric absorbance of each sample was measured at 490 nm using a microplate reader (BioTek Instruments, Inc., Winooski, VT). Cell viability was expressed as percentage of the vehicle control.

### 5-Ethynyl-2'-Deoxyuridine (EdU) Assay

Cell proliferation was detected using an EdU kit following the manufacturer’s instructions. HUVECs were seeded at a density of 2 × 10^5^ per well in 6-well plates and grown overnight. The cells were exposed to the vehicle or FMNT (10, 20 and 40 μM) for 24 h or pretreated with L-NAME (100 μM), LY294002 (20 μM), or PD98059 (10 μM) for 1 h before incubation with FMNT (20 μM) for 24 h with VEGF (40 ng/ml) as positive control. After incubation with EdU-labeling medium (10 μM) for 24 h, the cells were fixed with 4% paraformaldehyde for 30 min, and permeabilized with 0.5% Triton X-100 for 20 min. Then, the cells were incubated with Click-iT^®^ reaction cocktails for 30 min, washed with PBS, and stained with Hoechst 33342 dye (5 μg/ml) for 30 min. The images were examined and analyzed using a fluorescence microscope (Leica, Germany). The percentages of EdU-positive cells were calculated by Image J software.

### Cell Apoptosis Assay

Apoptosis was assessed using Annexin V-fluorescein isothiocyanate (FITC)/propidium iodide (PI) dual staining detection kit as per manufacturer’s instructions (BD Biosciences, Oxford, UK). HUVECs were seeded at a density of 2 × 10^5^ per well in 6-well plates and treated with the vehicle or FMNT (60, 80 and 100 μM) for 24 h. Then, the cells were washed with cold PBS twice, and stained with AnnexinV-FITC and PI in binding buffer for 15 min at room temperature in dark. Stained cells were quantified by flow cytometry (BD Biosciences, San Jose, CA). The data were analyzed by Flow Jo 7.6.1 software (Tree Star, Inc., Ashland, OR).

### Wound-Healing and Transwell Migration Assays

HUVECs were seeded at a density of 2 × 10^5^ per well in 6-well plates and exposed to the vehicle or FMNT (10, 20 and 40 μM) for 24 h or pretreated with L-NAME (100 μM), or LY294002 (20 μM) or PD98059 (10 μM) for 1 h before incubation with FMNT (20 μM) for 24 h. Cells treated with VEGF (40 ng/ml) served as positive control. After the treatment, scratch lines were created using 1000 μl pipette tips. The media was immediately replaced with fresh media in the absence of FMNT and VEGF. Photomicrographs of the scratch lines were taken using a microscope (Moticam 5+) at 100× magnification at the time of initial creation and at 6 h and 9 h of culture. The initial and final scratch line sizes were measured using Image J software.

A cytoselect 24-well cell migration kit (Cell Biolabs, Inc., San Diego, CA) was used for further migration evaluation according to manufacturer’s protocol. In brief, cell suspension containing 2.0 × 10^5^ cells/well in FBS-free medium was prepared. A 300 μl of cell suspension containing FMNT (10, 20 and 40 μM), or FMNT (20 μM) combined with L-NAME (100 μM), or LY294002 (20 μM) or PD98059 (10 μM) was added to each chamber. Cells treated with VEGF (40 ng/ml) served as a positive control. A 500 μl of complete medium was added to the lower chamber of the migration plate. The cells were incubated with the test compounds at 37˚C for 24 h. Cells migrating to the other side of the chamber were stained and examined using a microscope (Moticam 5+) at 200× magnification, with at least three individual fields per chamber. The stained cells were counted using Image J software.

### Tube Formation Assay

*In vitro* tube formation was performed with Matrigel Matrix 356237 (Corning Inc., Life Sciences, MA) as per manufacturer’s recommendation. 96-well plates were precoated with Matrigel (50 μl/well), which was allowed to solidify at 37°C for 60 min. HUVECs were exposed to the vehicle or FMNT (10, 20 and 40 μM) for 24 h or pretreated with L-NAME (100 μM), or LY294002 (20 μM) or PD98059 (10 μM) for 1 h before incubation with FMNT (20 μM) for 24 h. Cells cultured with VEGF (40 ng/ml) were used as a positive control. After the treatment, cells were harvested, resuspended and plated on Matrigel (20,000 cells/well) at 37°C for additional 3 h or 5 h. Quantification of the tubes was performed by analyzing three images of each well with a microscope (Moticam 5+) at 100× magnification, and the closed networks of vessel-like tubes were counted in each image with the average to be used for data analysis. The experiment was repeated at least three times to determine tube formation for each treatment group. The tube formation was expressed as the percentage over the vehicle control.

### NO Assay

Intracellular NO level was detected using a NO indicator DAF-FM diacetate following the manufacturer’s instructions. In brief, HUVECs were exposed to the vehicle or FMNT (10, 20 and 40 μM) for 24 h or pretreated with L-NAME (100 μM) or LY294002 (20 μM) or PD98059 (10 μM) for 1 h before incubation with FMNT (20 μM) for 24 h. After the treatment, cells were washed with PBS and incubated with DAF-FM diacetate (10 μM) for additional 1 h at 37°C. The cells were collected and the intracellular fluorescence intensity of DAF-FM was measured by a flow cytometer (BD Biosciences, San Jose, CA) with a 488 nm argon laser and FL1 channel, and analyzed with Flow Jo 7.6.1 software (Tree Star, Inc., Ashland, OR). The mean fluorescence value was converted to the percentage of control. The fluorescence intensity of DAF-FM in the cells was also evaluated with a confocal fluorescence microscope (Leica, Germany). After treatment and preparation, the cells were washed with PBS and fixed in 4% paraformaldehyde for 30 min. After washing with PBS, the preparations were incubated with phalloidin (0.165 μM) for 30 min. After three washes with PBS, the intracellular fluorescence of DAF-FM was detected by a confocal fluorescence microscope (excitation wave length = 488 nm; emission wave length = 535 nm).

### Western Blot Assay

HUVECs were exposed to the vehicle, FMNT (10, 20 and 40 μM) for 24 h or pretreated with L-NAME (100 μM), LY294002 (20 μM) and PD98059 (10 μM) for 1 h before incubation with FMNT (20 μM) for 24 h. At the end of treatment, proteins were prepared from the cells or mouse tissue samples from the healed skin using RIPA buffer containing a protease inhibitor cocktail and a phosphatase inhibitor cocktail (Thermoscientific, Rockford, IL). Protein concentrations were determined with a BCA estimation kit (Thermoscientific, Rockford, IL) according to the manufacturer’s instructions. Western blotting was performed as described (Wu et al., 2014). Briefly, protein (20 μg) was loaded onto each lane and separated by SDS-PAGE. Separated proteins were transferred from the gel to a PVDF membrane. After blocking for 2 h with non-fat milk (5%, w/v) in Tris-buffered saline, the primary antibodies of p-eNOS, eNOS, p-Akt, Akt, p-Erk1/2, Erk1/2 or GAPDH at 1:1,000 dilution was added to TBST with 5% nonfat milk, and incubated with the membrane at 4°C overnight. The membrane was washed and then incubated with corresponding secondary antibody at a dilution of 1:3,000 in blocking buffer for 1 h at room temperature. Western blot signals were obtained using an ECL chemiluminescence detection agent (Thermoscientific, Rockford, IL) following the manufacturer’s instructions. The relative intensity of each protein band was scanned and quantified using Quantity One software (Bio-Rad, Hercules, CA).

### Immunofluorescence Assay

HUVECs with different treatments were fixed with 4% paraformaldehyde for 30 min and then permeabilized with 0.5% Triton X-100 for 20 min. After being blocked with 0.5% bovine serum albumin, the cells were incubated with monoclonal anti-p-Akt or anti-p-Erk1/2 antibody (1:1000) at 4°C overnight. The cells then stained with fluorescent secondary antibody (1:400; Alexa Fluor 488, Invitrogen, Carlsbad, CA) for 1 h. Cells were then washed with PBS and incubated with phalloidin (0.165 μM) for 30 min, and DAPI (5 μ g/ml) for 20 min, respectively. The preparations were examined using a confocal microscope (Leica, Germany). The relative fluorescence of anti-p-Akt or anti-p-Erk1/2 was analyzed with ImageJ software.

### siRNA Interference

siRNA interference was performed using a Lipofectamine™ 3000 Transfection Kit according to the manufacturer's instructions. Briefly, siRNA and Lipofectamine™ 3000 were diluted in Opti-MEM medium, and mixed. The mixture was incubated at room temperature for 15 min to form a transfection complex, and then added to the cell culture medium for transfection. The control siRNA sequences were 5′-CGU​ACG​CGG​AAU​ACU​UCG​A-3′ (sense) and 5′-UCG​AAG​UAU​UCC​GCG​UAC​G-3′ (antisense). The Erk1/2 siRNA sequences were 5′-CCU​CCA​ACC​UGC​UCA​UCA​A-3′ (sense) and 5′-UUG​AUG​AGC​AGG​UUG​GAG​G-3′ (antisense). The Akt siRNA sequences were 5′-UGC​CCU​UCU​ACA​ACC​AGG​A-3′ (sense) and 5′-UCC​UGG​UUG​UAG​AAG​GGC​A-3′ (antisense). The cells were transfected with 50 nM Erk1/2-targeting siRNA (siErk1/2), Akt-targeting siRNA (siAkt) or control siRNA (siCon). At 48 h after transfection, the cells were used to repeat the experiments described above. At the end of treatment, the cells were collected for Western blot analysis, NO measurement, migration assay, tube formation assay or CCK8 assay as described above.

### Data Analysis

The data are expressed as mean ± standard deviation (SD), and analyzed using unpaired Student’s *t*-test or one-way ANOVA (analysis of variance) followed by post-hoc test by SPSS 19.0. A *p* value of < 0.05 was considered to be statistically significant.

## Results

### Prediction Analysis of the Pharmacological Targets and Mechanisms Based on Network Pharmacology

**FIGURE 1:**
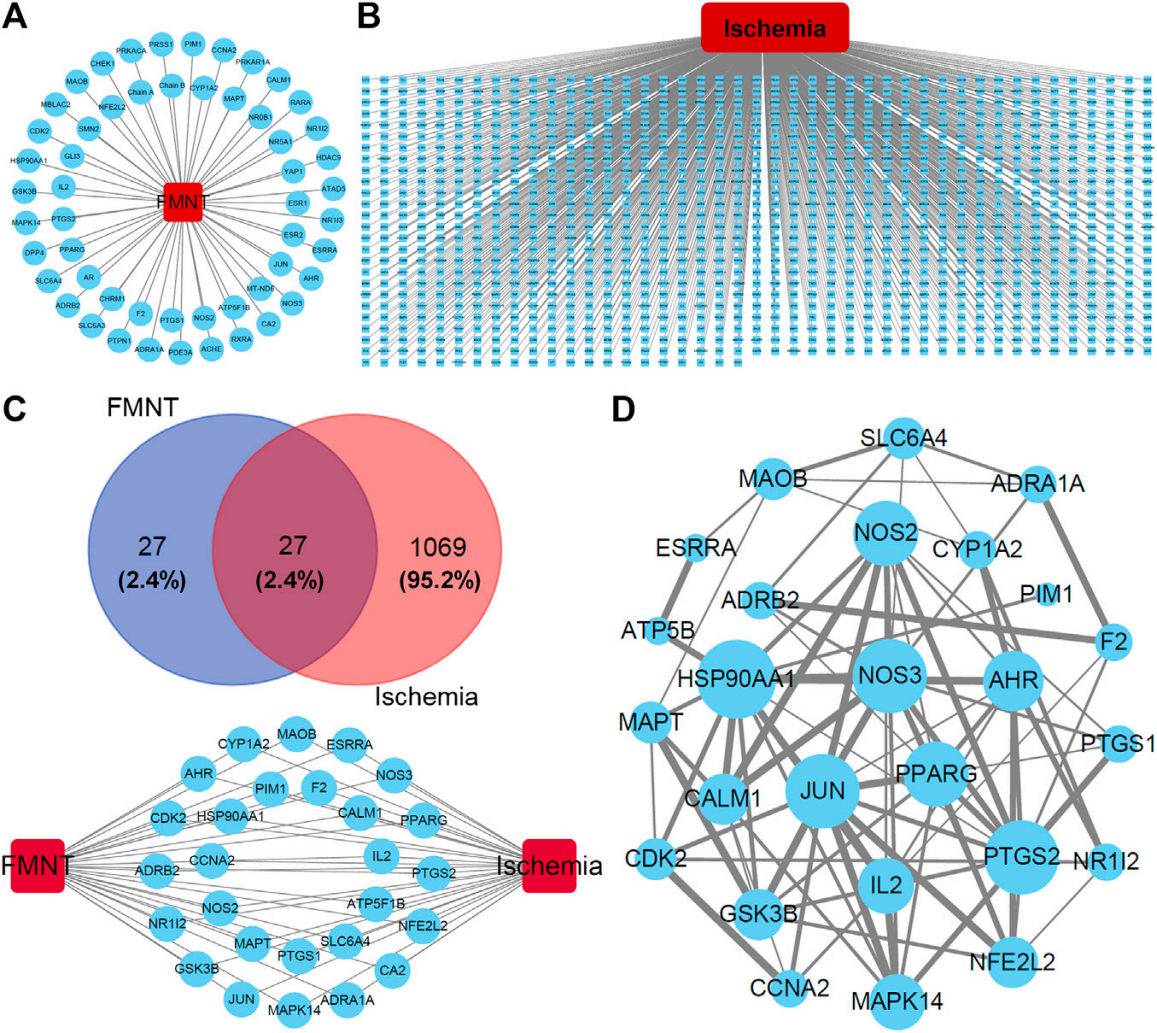
Potential targets and their interactions for the effect of FMNT against ischemia. **(A)** Potential target network for FMNT. **(B)** Potential network involved in ischemia. **(C)** Common targets for both FMNT and ischemia. **(D)** Interaction network of the common targets for FMNT and ischemia. Blue nodes represented the targets with their sizes proportional to the degree centrality determined with topology analysis. Edge size represented the combined score. The data were from STRING database and produced with Cytoscape 3.7.2 software.

**FIGURE 2:**
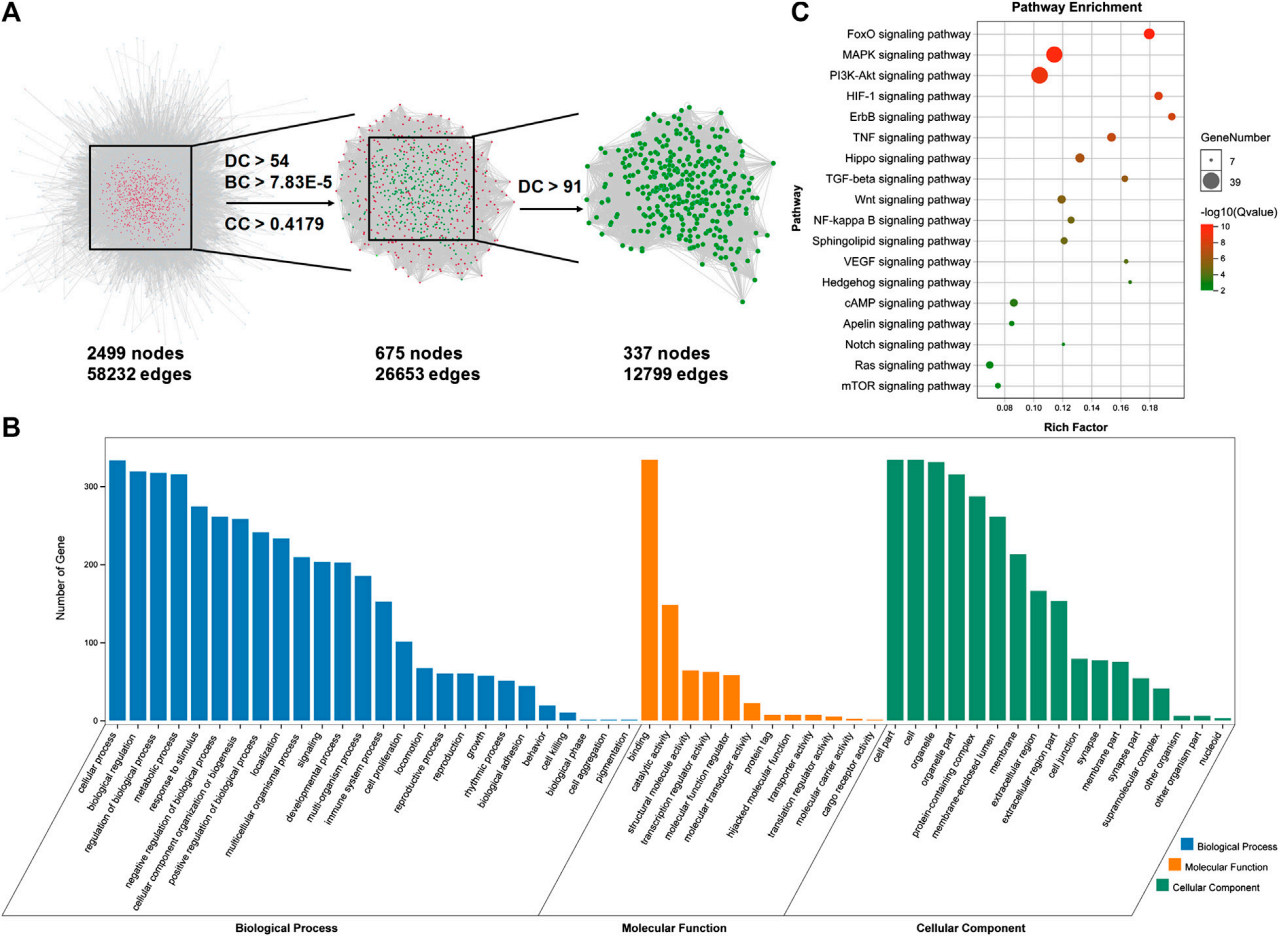
Identification and analysis of potential targets for FMNT against ischemia. **(A)** Identification of candidate FMNT targets for ischemia treatment through PPI network with topology analysis. A total of 337 candidate targets were predicted with the model. **(B)** GO enrichment analysis of the candidate targets for FMNT against ischemia. **(C)** KEGG enrichment analysis of candidate targets for FMNT against ischemia.

After analyzing the databases of TCMSP (http://tcmspw.com/index.php) and PubChem (https://pubchem.ncbi.nlm.nih.gov) (Hua et al., 2019; Zhu et al., 2019), a total of 54 potential pharmacological targets were identified for FMNT and the target network was visualized by Cytoscape 3.7.2 software (Gan et al., 2019; Zhu et al., 2019) (Figure 1A). Additionally, a group of 1096 genes associated with ischemia were identified through the CTD database (http://ctdbase.org) (Gan et al., 2019) (Figure 1B). 27 potential targets of FMNT were associated with ischemia (Figure 1C). The protein–protein interaction (PPI) network of the 27 common targets were obtained from the STRING database and visualized by Cytoscape 3.7.2 software (Figure 1D). The PPI network of the 27 FMNT targets was further established using the Cytoscape 3.7.2 software based on BisoGenet (Gan et al., 2019; Hua et al., 2019). The topological properties of each node in the network were assessed using the NetworkAnalyzer plugin. There were 2499 nodes and 58232 edges in the network with a medium degree value of 27 (Figure 2A). Three topological features, including betweenness centrality (BC), degree centrality (DC), and closeness centrality (CC), were selected to identify candidate targets, and 337 candidate targets were identified (Figure 2A).

Gene Ontology (GO) analysis and KEGG pathway enrichment analysis were performed using the online functional annotation and enrichment tool DAVID (https://david.ncifcrf.gov/) (Gan et al., 2019). GO terms and KEGG pathways with a *p* value < 0.05 were considered statistically significant. GO analysis showed that the majority of these 337 targets were enriched in cells and organelle with molecular function of protein binding (Figure 2B). Specifically, these targets were abundantly enriched in cellular process including cell metabolism and response to stimuli. These information implicated that various cellular functions could be involved in the diverse and synergistic effects of FMNT against ischemia. In addition, various signaling molecules, such as FoxO, MAPK, PI3K-Akt, HIF-1, ErbB, TNF, Hippo, TGF-beta, Wnt, NF-kappa B, Sphingolipid, VEGF, Hedgehog, cAMP, Apelin, Notch, Ras and mTOR signaling pathways (*Q* value < 0.05) involved in the signal transduction, were identified using KEGG pathway enrichment analysis (Figure 2C).

### Biphasic Effect of FMNT on Growth and Proliferation of HUVECs

**FIGURE 3:**
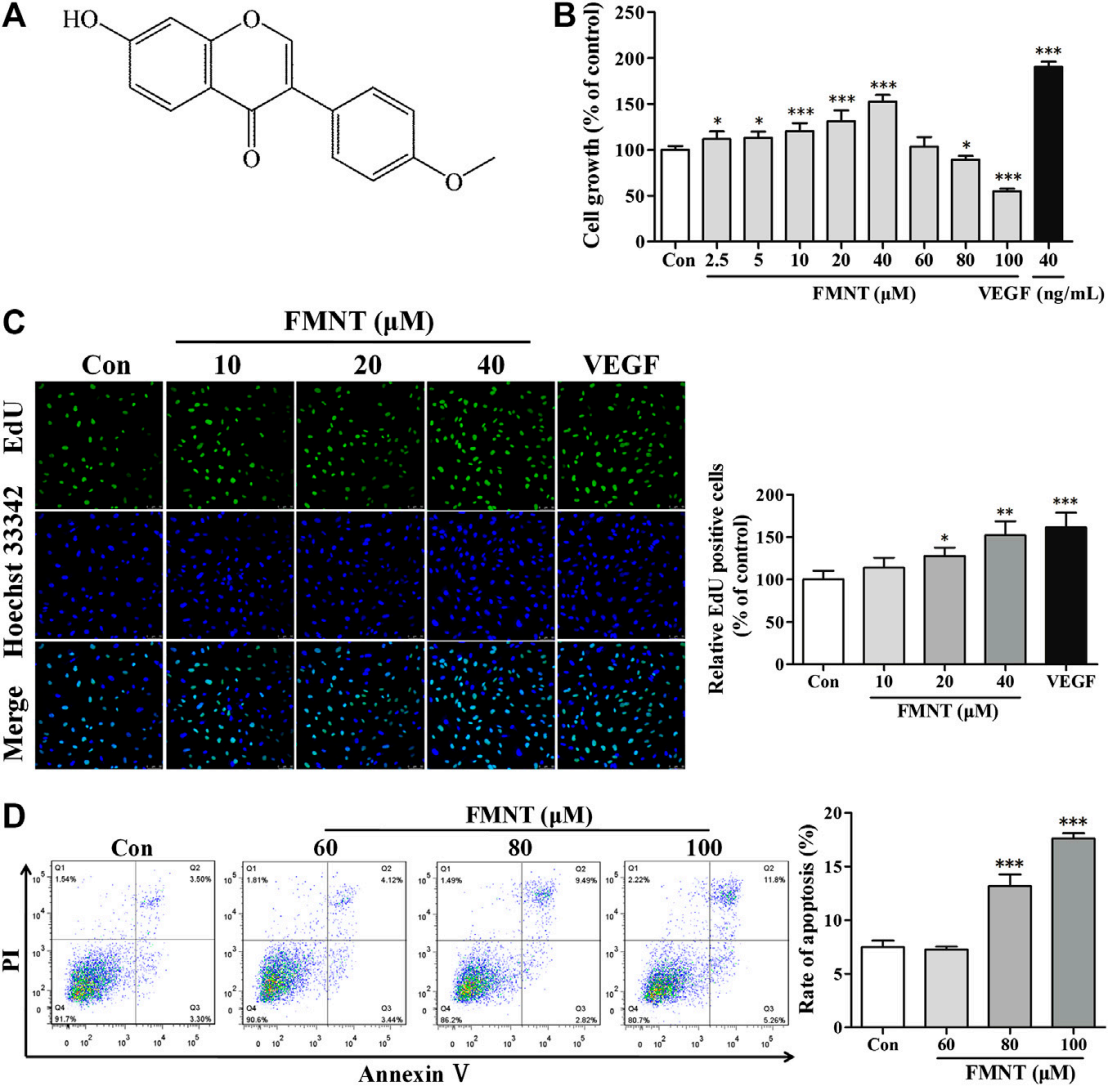
Effects of FMNT on the growth and proliferation of HUVECs. **(A)** Chemical structure of FMNT. **(B)** Cytotoxic effect of FMNT (2.5–100 μM, 24 h) on HUVECs was evaluated by using a CCK8 assay. **(C)** Effects of FMNT (10, 20, and 40 μM, 24 h) on the proliferation HUVECs was evaluated using an EdU kit. The EdU-positive cells in each group were quantified as the percentage of the cells in control group. **(D)** Effects of FMNT (60, 80, and 100 μM, 24 h) on the apoptosis of HUVECs was assessed using an Annexin V-FITC/PI dual staining detection kit. VEGF (40 ng/ml) was used as a positive control for endothelial growth and proliferation. The data were presented as mean ± SD (*n* = 3). ^*^ *p* < 0.05, **p < 0.01, and ***p < 0.001 compared with the control group.

FMNT (C_16_H_12_O_4_) is a member of the class of 7-hydroxyisoflavones with substitution by a methoxy group at position 4' with a molecular weight of 268.26 as shown in Figure 3A (Lou et al., 2019). The effect of FMNT on endothelial cell growth was evaluated using CCK8 assay. Figure 3B showed the dose-dependent effect of FMNT (2.5–100 μM) on HUVECs after 24 h of treatment. Compared with the control group, treatment with FMNT at 2.5–40 μM significantly increased cell growth dose-dependently by up to 52.52% (*p* < 0.05). However, when HUVECs were treated with FMNT at 80 or 100 μM, cell growth was significantly inhibited by 10.45% (*p* < 0.05) and 45.00% (*p* < 0.001), respectively. As expected, treatment with VEGF (40 ng/ml) markedly increased cell growth. Cell proliferation was determined using an EdU kit. As shown in Figure 3C, treatment with FMNT at 10, 20, and 40 μM for 24 h significantly increased EdU positive cells by 13.77%, 27.78% (*p* < 0.05) and 52.02% (*p* < 0.001), respectively, over the control. VEGF treatment (40 ng/ml) also increased HUVECs proliferation as expected (*p* < 0.001).

Annexin V-FITC/PI dual staining assay demonstrated that basal level of apoptosis for HUVECs was 7.49 ± 0.61%, while the apoptosis of the cells exposed to 60, 80, and 100 μM for 24 h was significantly increased to 7.26 ± 0.27%, 13.17 ± 1.095 (*p* < 0.001) and 17.61 ± 0.48% (*p* < 0.001), respectively (Figures 3D). Based on these results, three concentrations of 10, 20, and 40 μM, and a duration of 24 h treatment were used to determine the effect of FMNT on angiogenesis.

### FMNT Enhances Migration and Tube Formation of HUVECs

**FIGURE 4:**
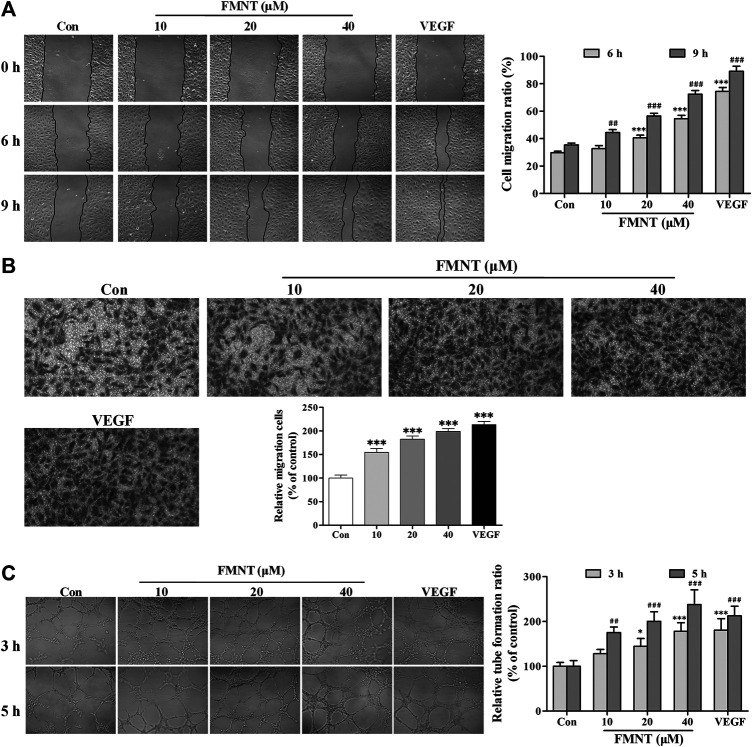
FMNT enhanced the migration and tube formation of HUVECs. **(A)** The effect of FMNT on HUVECs migration was examined using a wound healing method. HUVECs were pretreated with FMNT at 10, 20, and 40 μM for 24 h. Representative photomicrographs of the initial and final wounds at 6 and 9 h were obtained at 100× magnification. **(B)** Transwell migration assay was preformed to confirm the impact of FMNT on HUVECs migration. HUVECs were treated with FMNT at 10, 20, and 40 μM for 24 h. Cells migrating to other sides of the inserts were stained and examined using a microscope at 200× magnification. **(C)** The effect of FMNT on *in vitro* tube formation by HUVECs was assessed using a matrigel assay. HUVECs were pretreated with FMNT at 10, 20, and 40 μM for 24 h. After the treatment, the cells were plated on matrigel for additional 3 h or 5 h. Quantification of the tubes was performed by taking three images of each well with a microscope at 100 × magnification, then the closed networks of vessel-like tubes were counted in each image and analyzed. The data on tube formation were expressed as the percentage of the vehicle control. VEGF (40 ng/ml) was used as a positive control for endothelial migration and tube formation. The data were presented as mean ± SD (*n* = 3). ^*^ *p* < 0.05, and ***p < 0.001 or ^##^ *p* < 0.01, and ^###^ *p* < 0.001 compared with the corresponding control groups.

The effect of FMNT on migration of HUVECs was examined using wound healing and transwell migration. As shown in Figure 4A, pretreatment with FMNT at 10, 20, and 40 μM for 24 h significantly promoted HUVECs migration ratio by 10.26%–104.07% dose-dependently over the control (*p* < 0.001). Transwell migration assay demonstrated that treatment with FMNT at 10, 20, and 40 μM for 24 h significantly increased cell migration by 54.57% (*p* < 0.001), 82.60% (*p* < 0.001) and 99.01% (*p* < 0.001), respectively (Figure 4B), compared to the control.

Matrigel tube formation assay showed that little complete capillary-like structure formed by HUVECs without VEGF or FMNT treatment. In the presence of VEGF, capillary-like structure formation was significantly increased (*p* < 0.001). Similarly, pretreatment with FMNT at 10, 20, and 40 μM for 24 h significantly stimulated capillary-like structure formation by 27.78% and 137.50%, respectively, (*p* < 0.05, *p* < 0.01 or *p* < 0.001). The capillary-like structure formation by the cells treated with 40 μM FMNT was comparable to that with 40 ng/ml VEGF (Figure 4C).

### FMNT Increased eNOS Phosphorylation and Intracellular NO Level in HUVECs

**FIGURE 5:**
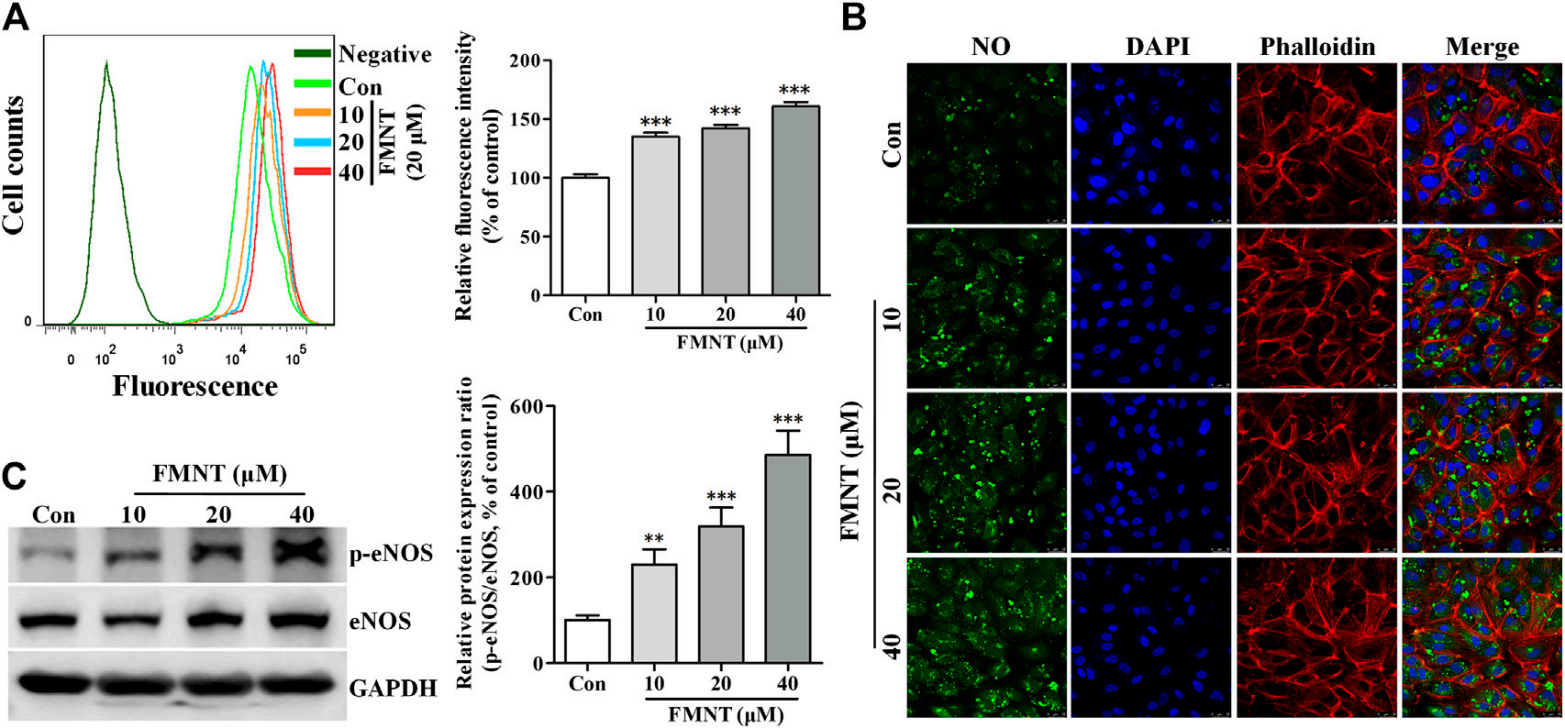
FMNT induced eNOS phosphorylation with increased intracellular NO levels in HUVECs. HUVECs were treated with FMNT at 10, 20, and 40 μM for 24 h. Intracellular NO levels were detected using a NO indicator DAF-FM diacetate. Intracellular fluorescence intensity of DAF-FM was determined with a flow cytometer **(A)** and a confocal fluorescence microscope **(B)**. The mean fluorescence value was converted to the percentage of control. **(C)** The effect of FMNT on eNOS phosphorylation in HUVECs were evaluated with Western blot analysis. The protein expression data were expressed as the percentage of control. The data were presented as mean ± SD (*n* = 3). ^*^ *p* < 0.05, **p < 0.01, and ***p < 0.001 compared with the control group.

Flow cytometry analysis showed that intracellular NO level as reflected by intracellular fluorescence intensity of DAF-FM was significantly increased in the cells treated with FMNT at 10, 20, and 40 μM for 24 h by 34.85% (*p* < 0.001), 42.11% (*p* < 0.001) and 60.80% (*p* < 0.001) over the control, respectively (Figure 5A). Confocal fluorescence microscope analysis also showed that the fluorescence intensity of DAF-FM was significantly increased in the cells treated with FMNT over control cells (Figure 5B). Person correlation analysis showed that increased NO production induced by FMNT was closely related to cell growth (Supplementary Figure S1B), proliferation (Supplementary Figure S1C), migration (Supplementary Figures S1D,E), and tube formation (Supplementary Figure S1F).

Western blot analysis showed that the protein level of eNOS phosphorylation (p-eNOS) was significantly increased in HUVECs treated with FMNT in a dose dependent manner (*p* < 0.001, Figure 5C). Immunofluorescence assay further confirmed the level of p-eNOS in the cells treated with FMNT (Supplementary Figure S2A). Total eNOS protein expression was also significantly increased in the cells (Figure 5C, *p* < 0.05 or *p* < 0.01). The p-eNOS/total eNOS ratio was 2.30- (*p* < 0.01), 3.19- (*p* < 0.001), and 4.86-fold (*p* < 0.001) higher in the cells treated with 10, 20, and 40 μM FMNT for 24 h, respectively. A strong positive correlation was also observed between the increase in p-eNOS/eNOS ratio and increase in NO level in the cells treated with FMNT (Supplementary Figure 2B, *r* ^2^ = 0.9276. *p* < 0.05).

### FMNT Activated Erk1/2 and Akt Pathways in HUVECs

**FIGURE 6:**
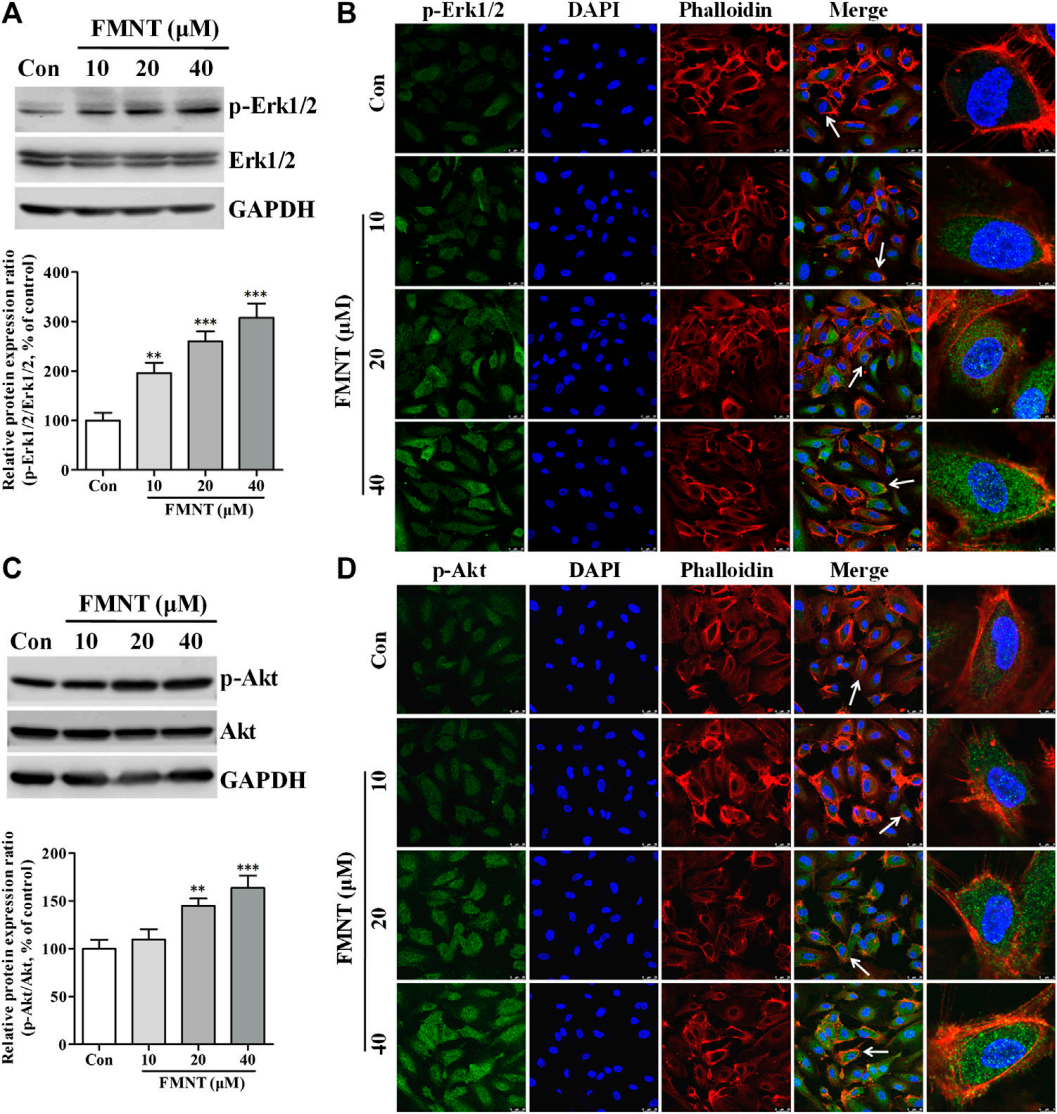
FMNT activated Erk1/2 and Akt pathways in HUVECs. HUVECs were treated with FMNT at 10, 20, and 40 μM for 24 h. The effects of FMNT on Erk1/2 and Akt phosphorylation in HUVECs were evaluated with Western blot analysis **(A,C)** and immunofluorescence assay **(B,D)**. The protein expression and fluorescence value were expressed as the percentage of control. The data were presented as mean ± SD (*n* = 3). **p < 0.01, and ***p < 0.001 compared with the control group.

Western blot analysis (Figure 6A) showed that treatment with FMNT at 10, 20, and 40 μM significantly increased the protein level of p-Erk1/2 in the cells in a dose dependent manner (*p* < 0.001), without significant change in total Erk1/2. The p-Erk1/2**/**Erk1/2 ratio was 1.96- (*p* < 0.01), 2.61- (*p* < 0.001), and 3.08-fold (*p* < 0.001) higher in the cells treated with 10, 20, and 40 μM FMNT over the control, respectively. Immunofluorescence assay also demonstrated an increased level of Erk1/2 phosphorylation (p-Erk1/2) in the cells treated with FMNT dose-dependently (Figure 6B). FMNT treatment also significantly increased the protein level of p-Akt (Figure 6C, *p* < 0.01) without significantly altering the total Akt protein expression (Figure 6C). Thus, p-Akt/Akt ratio was significantly increased by up to 63.54% (*p* < 0.001, Figure 6C). Similarly, immunofluorescence assay showed a significant increase in p-Akt expression in the cells with FMNT treatment (Figure 6D).

Person correlation analysis showed that FMNT-induced increase in p-Erk1/2/Erk1/2 ratio was closely related increased p-eNOS/eNOS ratio (Supplementary Figure S3B, *r* ^2^ = 0.9463. *p* < 0.05) and NO levels (Supplementary Figure S3C, *r* ^2^ = 0.9674. *p* < 0.05). A strong positive correlation was also observed between FMNT-induced increase in p-Erk1/2/Erk1/2 ratio and enhancement of cell growth, proliferation, migration, and tube formation (Supplementary Figure S3D–H). In addition, Person analysis revealed that FMNT-induced increase in p-Akt/Akt ratio was positively related to increased eNOS phosphorylation and NO production, as well as to enhanced angiogenesis by FMNT (Supplementary Figure S4).

### Erk1/2 and Akt Pathways Were Critically Involved in FMNT-Induced eNOS Phosphorylation and NO Production in HUVECs

**FIGURE 7:**
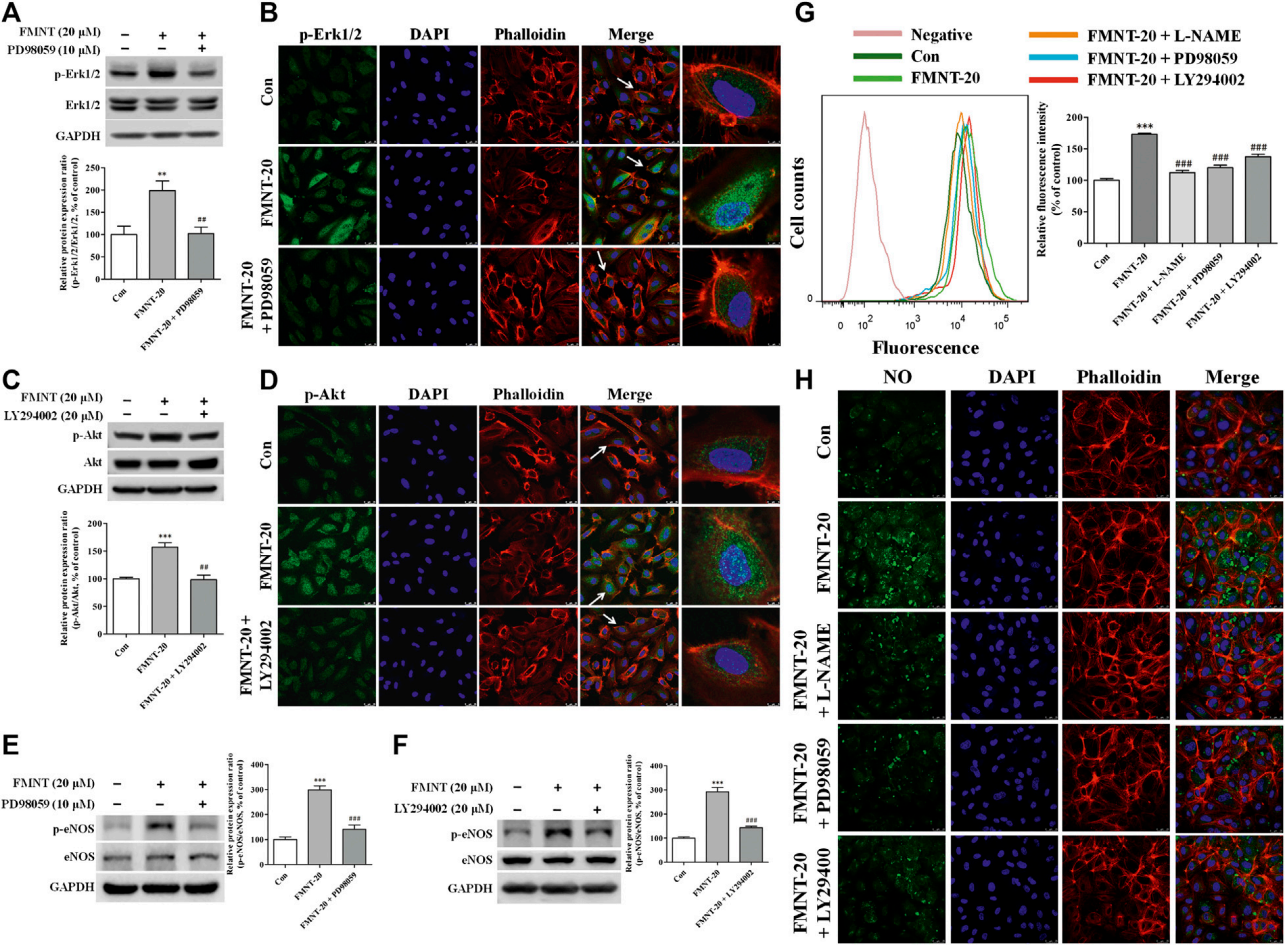
Specific inhibitors of Erk1/2 and Akt diminished FMNT-induced eNOS phosphorylation and NO production in HUVECs. Erk1/2 **(A,B)** and Akt **(C,D)** phosphorylation was detected with Western blot analysis and immunofluorescence assay in HUVECs exposed to FMNT (20 μM) for 24 h with and without pretreatment of PD98059 (10 μM) or LY294002 (20 μM) for 1 h eNOS phosphorylation was detected with Western blot analysis in HUVECs exposed to FMNT (20 μM) for 24 h with and without pretreatment of PD98059 (10 μM) **(E)** and LY294002 (20 μM) **(F)** for 1 h. Intracellular NO levels in HUVECs with the same treatments were detected using a NO indicator DAF-FM diacetate that was measured with a flow cytometer **(G)** and a confocal fluorescence microscope **(H)**. The protein expression and fluorescence value were expressed as the percentage of control. The data were presented as mean ± SD (*n* = 3). **p < 0.01, and ***p < 0.001 compared with the control group. ^##^ *p* < 0.01, and ^###^ *p* < 0.001 compared with the FMNT (20 μM) group.

To investigate the role of Erk1/2 and Akt pathways in mediating FMNT-induced eNOS phosphorylation and NO production in HUVECs, Erk1/2 and Akt phosphorylation by FMNT was attenuated using the specific inhibitors PD98059 and LY294002, respectively. Compared with the control, treatment with FMNT alone at 20 μM significantly increased p-Erk1/2**/**Erk1/2 ratio (Figure 7A, *p* < 0.01). FMNT-induced Erk1/2 phosphorylation was significantly decreased in the cells pre-treated with PD98059 as shown on Western blot analysis (Figure 7A) and immunofluorescence assay (Figure 7B). Similarly, pre-treatment with LY294002 effectively prevented FMNT-induced Akt phosphorylation in HUVECs (Figures 7C,D).

Compared with the cells exposed to FMNT alone, pre-treatment of the cells with PD98059 effectively prevented FMNT-induced protein expression of p-eNOS without significant change in the total eNOS in HUVECs (Figure 7E, *p* < 0.001), and thus p-eNOS**/**eNOS ratio was significantly decreased by 52.82% (Figure 7E, *p* < 0.001). Similar results were observed when the cells were pre-treated with LY294002 with marked decrease in p-eNOS**/**eNOS ratio by 50.89% over control (Figure 7F, *p* < 0.001). As expected, FMNT-induced production of intracellular NO was effectively blocked when the cells were pre-treated with L-NAME (a potent eNOS inhibitor), PD98059 or LY294002, by 35.31% (*p* < 0.001), 30.73% (*p* < 0.001) and 20.61% (*p* < 0.001), respectively (Figures 7G,H).

**FIGURE 8:**
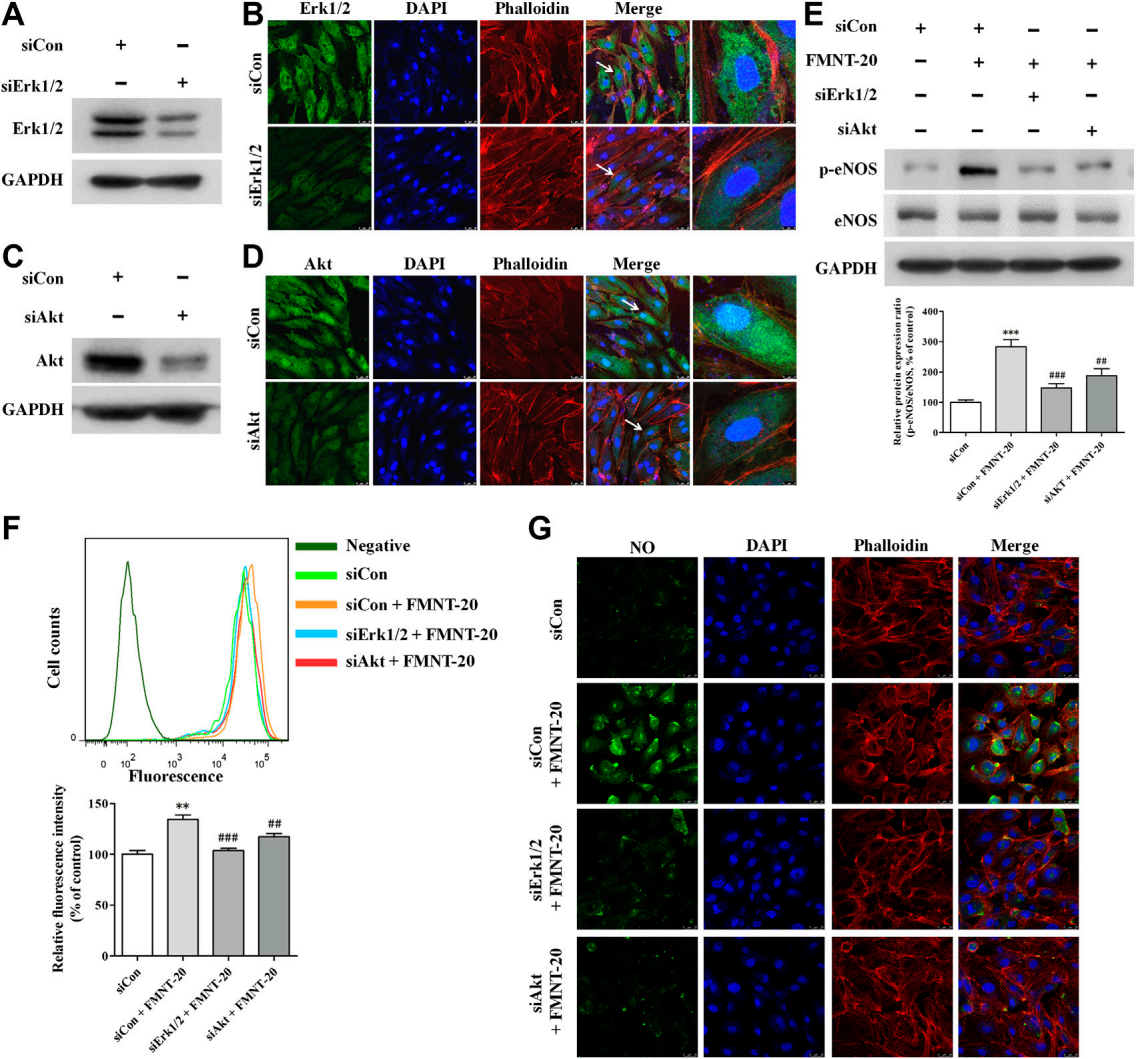
siErk1/2 and siAkt attenuated FMNT-induced eNOS phosphorylation and NO production in HUVECs. The expression of Erk1/2 and Akt was silenced in HUVECs with transfection with specific siRNA for Erk1/2 (siErk1/2) and for Akt (siAkt), respectively. Protein levels of Erk1/2 and Akt was detected with Western blot analysis and immunofluorescence assay, respectively **(A–D)**. **(E)** eNOS phosphorylation was detected with Western blot analysis in HUVECs with siErk1/2 or siAkt transfection. Intracellular NO levels in HUVECs with the same treatments were detected using a NO indicator DAF-FM diacetate with a flow cytometer **(F)** and a confocal fluorescence microscope **(G)**, respectively. The protein expression and fluorescence value were expressed as the percentage of control. The data were presented as mean ± SD (*n* = 3). **p < 0.01, and ***p < 0.001 compared with the control group. ^##^ *p* < 0.01, and ^###^ *p* < 0.001 compared with the FMNT (20 μM) group.

To further evaluate the role of Erk1/2 and Akt pathways in mediating the effect on FMNT-induced eNOS phosphorylation and NO production in HUVECs, the expression of Erk1/2 and Akt was silenced in HUVECs with transfection with Erk1/2-specific siRNA (siErk1/2) and Akt-specific siRNA (siAkt), respectively. As shown in Figures 8A–D, cells transfected with siErk1/2 and siAkt exhibited significant reduction in Erk1/2 and Akt expression compared with the cells transfected with control siRNA (siCon) (*p* < 0.001). In contrast to transfection with siCon, transfection with siErk1/2 or siAkt markedly attenuated FMNT-induced increase in p-eNOS**/**eNOS ratio (Figure 8E). It was also observed that FMNT-induced NO production was dramatically attenuated in the cells transfected with siErk1/2 or siAkt (Figure 8F, G).

### Erk1/2 and Akt Pathways Mediated the Effect of FMNT on Angiogenesis by HUVECs

**FIGURE 9:**
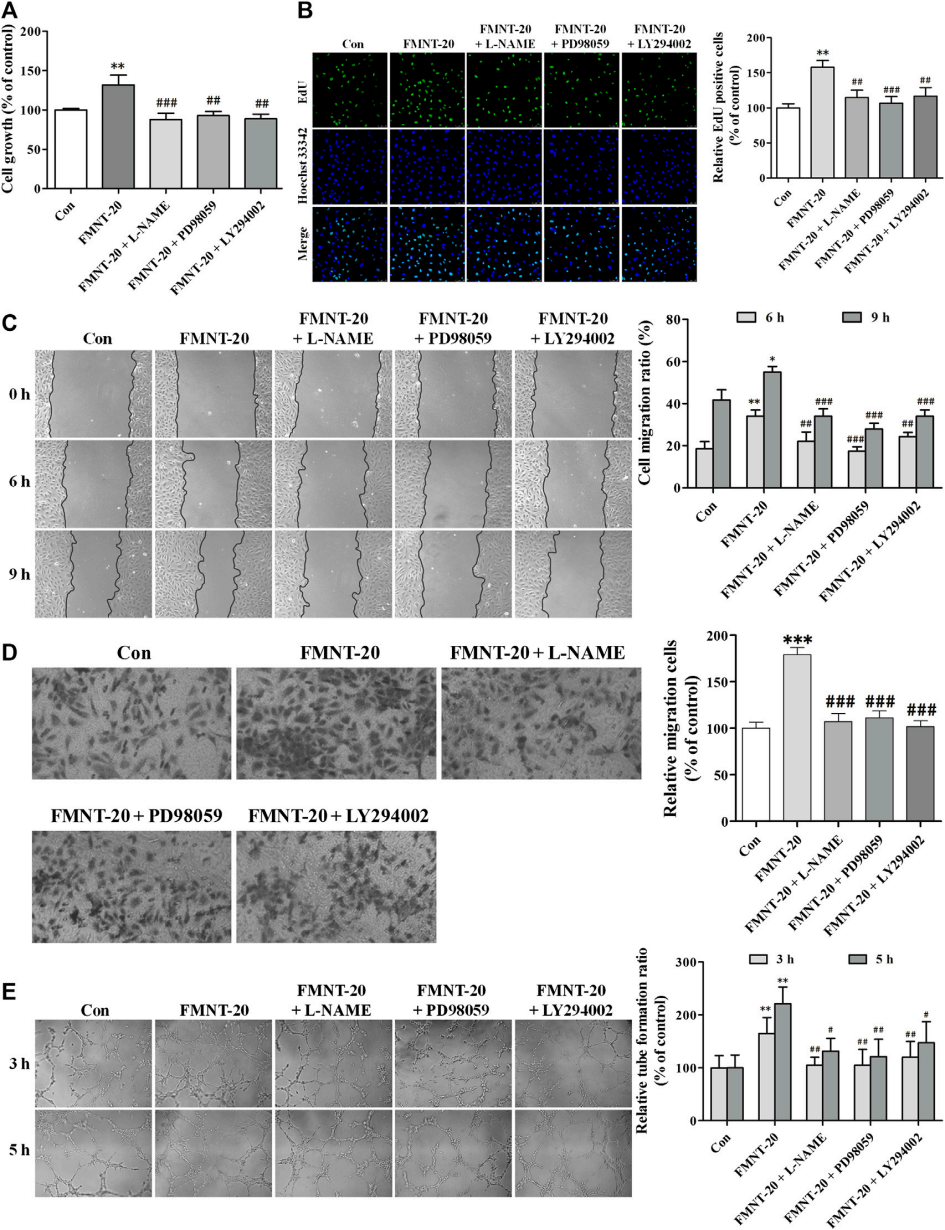
Specific inhibitors of Erk1/2 and Akt diminished the promotion of angiogenesis by FMNT in HUVECs. HUVECs are exposed to the FMNT (20 μM) for 24 h or pretreated with L-NAME (100 μM), PD98059 (10 μM) and LY294002 (20 μM) for 1 h before incubation with FMNT (20 μM) for 24 h. **(A)** Cell growth was assessed by using a CCK8 assay. **(B)** The ability of cell proliferation was detected by using an EdU kit. **(C)** The migration of HUVECs was examined using wound-healing **(C)** and transwell migration **(D)** assays, respectively. **(E)** The tube formation of HUVECs was assessed by using a matrigel assay. The data represent the mean ± SD (*n* = 3). ^*^ *p* < 0.05, **p < 0.01, and ***p < 0.001 compared with the control group. ^#^ *p* < 0.05, ^##^ *p* < 0.01, and ^###^ *p* < 0.001 compared with the FMNT (20 μM) group.

The role of Erk1/2- and Akt signaling-mediated activation of eNOS/NO pathway in mediating FMNT-induced enhancement of angiogenesis was evaluated using their respective specific inhibitors L-NAME, PD98059, and LY294002. As shown in Figure 9A, pre-treatment with L-NAME, PD98059, or LY294002 significantly attenuated FMNT-induced growth of HUVECs by 33.38% (*p* < 0.001), 29.60% (*p* < 0.01) and 32.76% (*p* < 0.01), respectively. EdU assay showed that inhibition of Erk1/2 and Akt, as well as eNOS/NO pathway with the specific inhibitors effectively blocked FMNT-induced increase in cell proliferation (Figure 9B, *p* < 0.01 or *p* < 0.001), cell migration (Figure 9C,D, *p* < 0.01 or *p* < 0.001), and tube formation (Figure 9E, *p* < 0.05 or *p* < 0.01).

**FIGURE 10:**
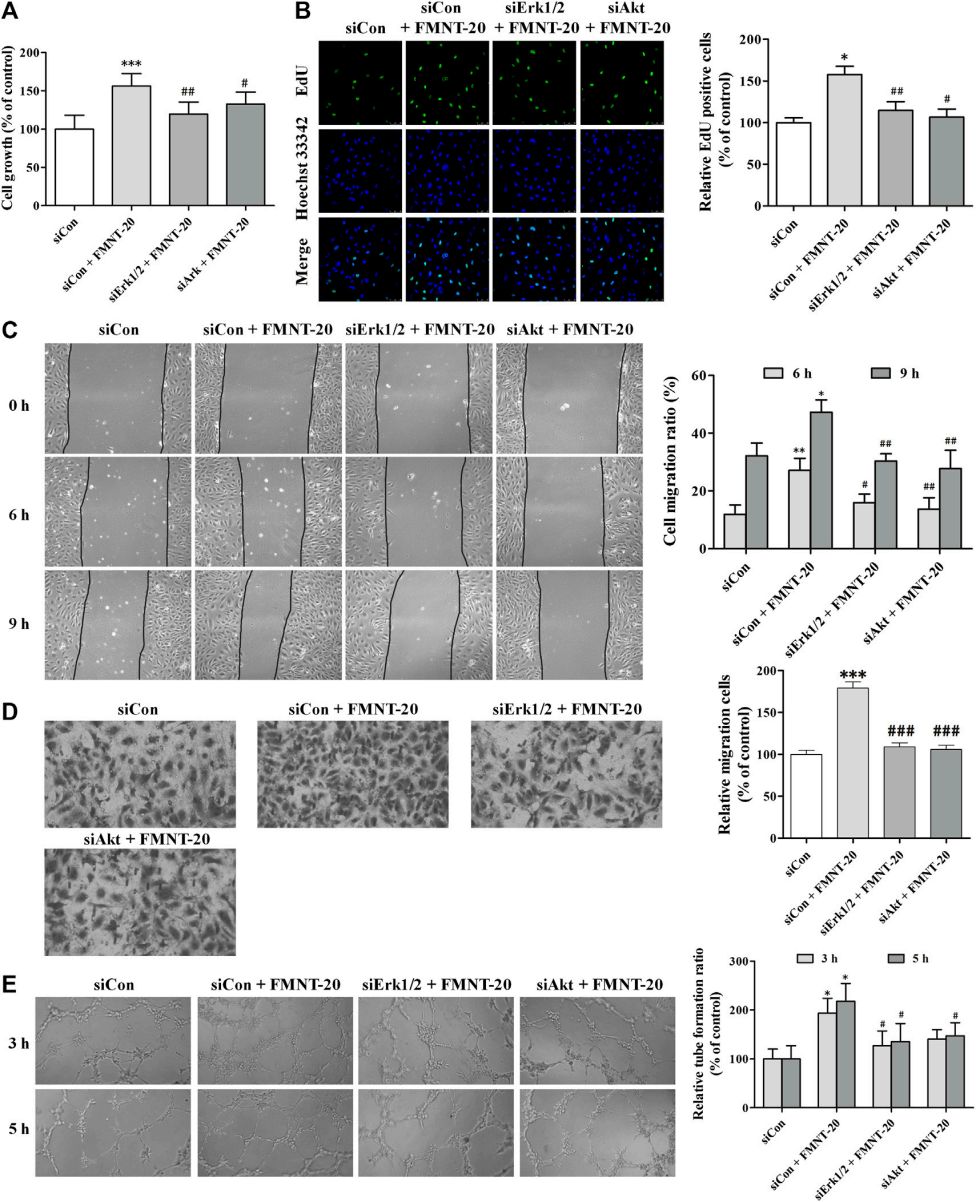
siErk1/2 and siAkt attenuated FMNT-induced angiogenesis in HUVECs. The expression of Erk1/2 and Akt was silenced in HUVECs with transfection with specific siRNA for Erk1/2 (siErk1/2) and Akt (siAkt), respectively. Then, HUVECs were exposed to FMNT (20 μM) for 24 h. **(A)** Cell growth was assessed using a CCK8 assay. **(B)** Cell proliferation was evaluated using an EdU kit. **(C)** The migration of HUVECs was examined using wound-healing **(C)** and transwell migration **(D)** assays, respectively. **(E)** Tube formation of HUVECs was assessed using a matrigel assay. The data were presented as mean ± SD (*n* = 3). ^*^ *p* < 0.05, **p < 0.01, and ***p < 0.001 compared with the control group. ^#^ *p* < 0.05, ^##^ *p* < 0.01, and ^###^ *p* < 0.001 compared with the FMNT (20 μM) group.

To further investigate the role of Erk1/2 signaling- and Akt signaling-mediated activation of eNOS/NO pathway in mediating FMNT-induced angiogenesis, expression of Erk1/2 and Akt was silenced in HUVECs with transfection with siErk1/2 and siAkt, respectively. Compared with the cells transfected with siCon and exposed to FMNT, cells transfected with siErk1/2 and siAkt and exposed to FMNT exhibited significant reductions in FMNT-induced cell growth (Figure 10A), cell proliferation (Figure 10B), migration (Figure 10C,D), and tube formation (Figure 10E).

### FMNT Enhanced Wound Healing in C57BL/6 Mice

**FIGURE 11:**
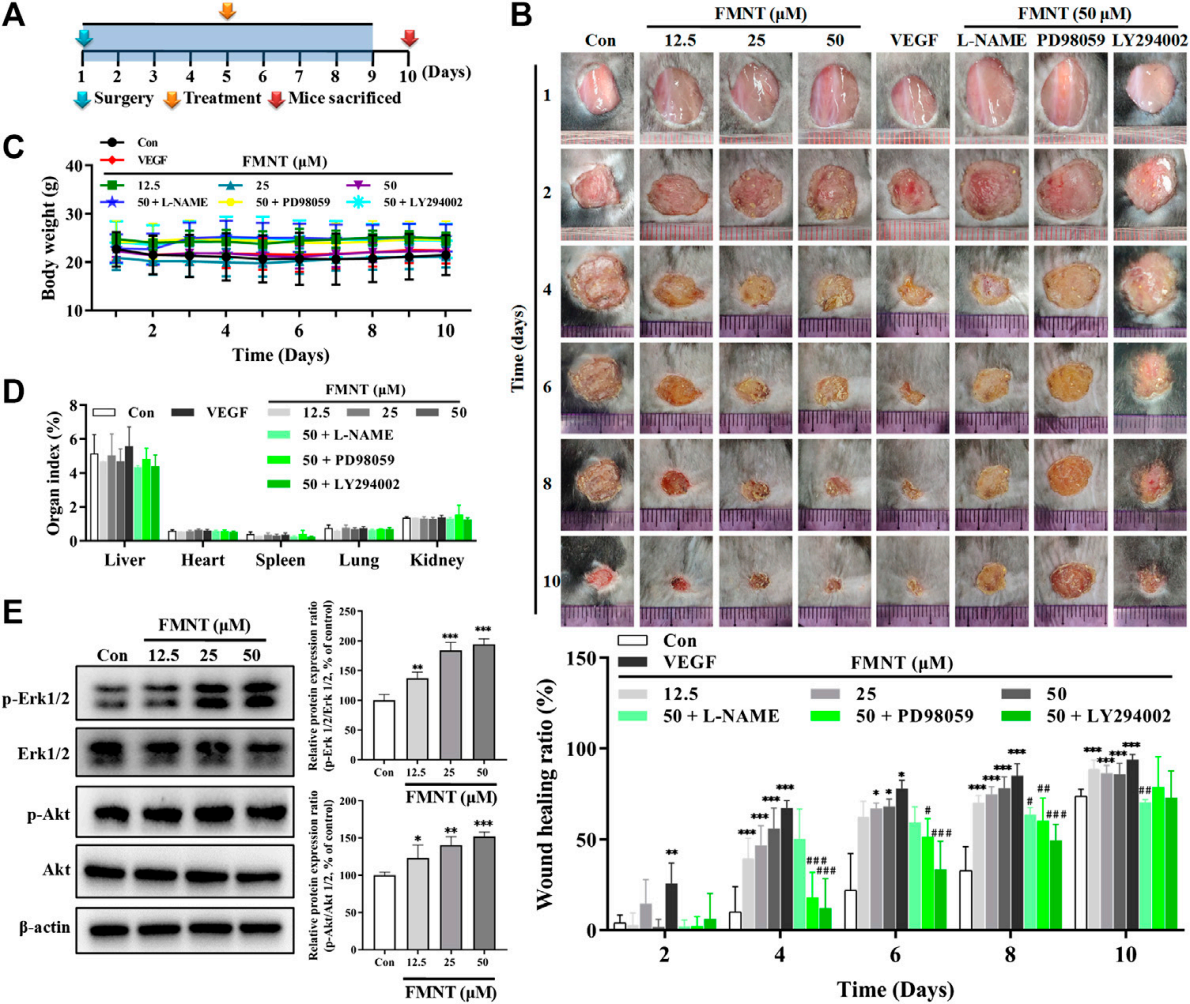
FMNT enhanced wound healing in a dermal wound model in mice. **(A)** A dermal wound model in mice was established to investigate the effects of FMNT on wound healing. **(B)** The wounds were photographed at postoperative days 2, 4, 6, 8, and 10. Wound area reduction was calculated as the percentage of the initial wound area. **(C)** Average body weight was recorded for the mice. **(D)** Organ indexes were calculated for mouse hearts, livers, spleens, kidneys and lungs. **(E)** The effects of FMNT on Erk1/2 and Akt phosphorylation in mice were evaluated with Western blot analysis. The data were presented as mean ± SD (*n* = 5 or 6). ^*^ *p* < 0.05, **p < 0.01, and ***p < 0.001 compared with the control group. ^#^ *p* < 0.05, ^##^ *p* < 0.01, and ^###^ *p* < 0.001 compared with the FMNT (50 μM) group.

A C57BL/6 mouse dermal wound healing model was established to investigate the effect of FMNT on wound healing (Figure 11A). As shown in Figure 11B, compared with the mice treated with the vehicle (PBS), treatment with FMNT at 12.5, 25, and 50 μM significantly accelerated the rate of wound closure as early as day 4 after surgery (Con = 10.2% ± 13.9%; FMNT at 12.5, 25, and 50 μM = 39.6% ± 11.0%, 46.8 ± 10.8% and 55.7% ± 12.6%; VEGF = 67.3 ± 4.1%). The effect of FMNT on reduction in wound area persisted at day 6, 8 and 10 after creation of wounding. FMNT-induced enhancement on wound healing was effectively attenuated when eNOS, Erk1/2 or Akt was inhibited by their respective specific inhibitors L-NAME, PD98059, and LY294002, respectively. Of note, there were no significant differences in body weight and organ indexes for heart, kidney, lung, liver and spleen between control mice and mice treated with FMNT for wound healing experiment (Figures 11C,D).

Western blot analysis showed that treatment with FMNT at 12.5, 25, and 50 μM significantly increased the protein levels of p-Erk1/2 in a dose dependent manner compared with the control, while no significant change was observed for the total Erk1/2. Thus, the p-Erk1/2**/**Erk1/2 ratio was 1.37- (*p* < 0.01), 1.84- (*p* < 0.001), and 1.94-fold (*p* < 0.001) higher in the mice treated with 12.5, 25, and 50 μM of FMNT, respectively. FMNT treatment also produced a significant increase in p-Akt protein without significant change in the total Akt protein. The p-Akt/Akt ratio was significantly increased by 23.06% (*p* < 0.05), 40.17% (*p* < 0.01) and 51.90% (*p* < 0.001) over the control, respectively (Figure 11E).

## Discussion

In the present study, we first predicted the potential targets of FMNT and mechanism for the pro-angiogenic effect of FMNT using a network pharmacology approach. A total of 27 potential targets of FMNT that were associated with ischemia were identified (Figure 1). Functional and pathway enrichment analysis revealed that multiple signaling molecules including MAPK and PI3K-Akt pathways could be involved in the pharmacological actions of FMNT against ischemic diseases (Figure 2). The data from our experimental study further demonstrated that FMNT promoted endothelial function and angiogenesis *in vitro* and *in vivo* in a PI3K/Akt and Erk1/2-dependent manner.

Previous studies showed that FMNT could promote early fracture healing through stimulating angiogenesis by up-regulating VEGFR-2/Flk-1 (Huh et al., 2009). FMNT could also promote endothelial repair and wound healing in association with over-expression of Egr-1 transcription factor and several growth factors (Huh et al., 2011). In addition, FMNT could induce endothelial cell migration and promote angiogenesis through estrogen receptor alpha-enhanced ROCK pathway (Li et al., 2015). However, it is not fully understood if FMNT could enhance eNOS activation with increased NO production, thus augmenting endothelial function. NO is generated endogenously from L-arginine in the presence of oxygen and NADPH by the enzyme eNOS (Lee et al., 2018). Phosphorylation of eNOS is critically involved in the regulation of eNOS activity and NO production, and is essential for endothelial function like angiogenesis (Lee et al., 2018). The data from the present study demonstrated that FMNT exhibited biphasic effects on endothelial function. At concentrations of 2.5–40 μM, FMNT could promote endothelial function through activation of eNOS/NO signaling pathway in a dose dependent manner (Figure 3–5). However, when the concentrations were increased to 60 μM or above, FMNT could significantly inhibit the cell viability and increase apoptosis of endothelial cells *in vitro* (Figure 3). The finding in the present study that there is a biphasic effect of FMNT on endothelial cells is consistent with the observation in a previous report that showed that at concentrations of 0–25 μM, FMNT could promote HUVEC growth. However, when the concentrations were increased to 50 μM or above, FMNT could significantly inhibit the viability of HUVECs (Li et al., 2015). While this is a very interesting finding, the mechanism(s) for the inhibitory effect of FMNT toward HUVECs at high concentrations is unclear at this point. It is possible that FMNT at high concentrations (60 μM or above) could suppress the activities of ROCK pathway, Akt or Erk1/2 signaling in endothelial cells, leading to inhibition of endothelial function. Further studies are needed to define the mechanism(s) for the biphasic effects of FMNT.

Akt and Erk1/2 signaling plays critical roles in regulating endothelial function and angiogenesis (Wang et al., 2013; Ahmad et al., 2018; Lee et al., 2018). Activation of Akt and Erk1/2 signaling could result in sustained production of NO through eNOS phosphorylation, thus enhancing endothelial function with increased cell survival, proliferation, tube formation, migration, and angiogenesis (Eliceiri et al., 1998; Kawasaki et al., 2003; Wang et al., 2013). It was found that FMNT significantly induced the expression of phosphorylated Erk1/2 (p-Erk1/2) and Akt (p-Akt) without significant changes in their total proteins (Figure 6). Inhibition of Erk1/2 or Akt signaling effectively attenuated the effect of FMNT on endothelial function, eNOS activation, NO production, and angiogenesis in HUVECs (Figure 7–10). It was further observed that FMNT significantly accelerated the rate of wound closure in a mouse dermal wound healing model with activation of Erk1/2 and Akt signaling. The effect of FMNT on wound healing in mice was also effectively attenuated by inhibition of eNOS, Erk1/2 and Akt signaling (Figure 11). These results indicate that both Erk1/2 and Akt signaling could potentially mediate the enhanced eNOS phosphorylation and NO production to promote endothelial function and angiogenesis by FMNT *in vitro* and *in vivo.*

One of the key findings in the present study was that a potential interdependence between ERK1/2 and Akt signaling was required for the pharmacological action of FMNT on the activation of eNOS/NO signaling and endothelial function. As shown in Figures 7–10, silence of ERK1/2 or Akt signaling could nearly abolish eNOS/NO activation, endothelial function and angiogenesis by FMNT in HUVECs. However, when Akt expression was knocked down with siAkt in HUVECs, FMNT treatment was still able to activate Erk1/2 signaling without activation of eNOS/NO signaling and promotion of endothelial function. Similarly, FMNT could activate Akt signaling with no change in eNOS/NO activation and endothelial function when Erk1/2 expression was inhibited with siErk1/2 in HUVECs (Supplementary Figure S5). These data suggested that simultaneous activation of Erk1/2 and Akt signaling was critical for FMNT-induced eNOS phosphorylation and enhanced endothelial function and angiogenesis. We further analyzed the potential interactions between Erk1/2 and Akt signaling using network pharmacology, and noticed that MAPK (including 38 targets) and PI3K-Akt (including 39 targets) signaling pathways were involved in the effects of FMNT against ischemia (Figure 2 and Supplementary Figure S6). There were 16 common targets of MAPK and PI3K-Akt signaling pathways, and close interactions were present among nine targets, including the key targets MAPK1 (Erk2), MAPK3 (Erk1) and Akt1 (Supplementary Figure S6). Thus, it is very likely that ERK1/2 and Akt signaling pathways could interdependently contribute to eNOS activation and NO production and endothelial function by FMNT. Further studies are needed to determine the mechanism(s) on the interactions between Erk1/2 and Akt signaling on eNOS activation in mediating the effect of FMNT on HUVECs.

## Conclusion

In summary, the present study demonstrated that FMNT significantly enhanced endothelial function and promoted angiogenesis *in vitro* and *in vivo* through activating Erk1/2- and Akt-mediated eNOS/NO signaling pathway. The data also suggested that simultaneous activation of Erk1/2 and Akt signaling was required for FMNT-induced eNOS phosphorylation and enhanced endothelial function. Results from the present study might provide support and evidence for the application of FMNT during the clinical treatment of conditions related to vascular insufficiency, such as ischemic heart diseases, wound healing, and diabetic vascular complications.

### DATA AVAILABILITY STATEMENT

The original contributions presented in the study are included in the article/Supplementary Material, further inquiries can be directed to the corresponding authors.

## Ethics Statement

The animal study was reviewed and approved by Guangzhou University of Chinese Medicine Animal Care and Use Committee (Guangzhou, China).

## Author Contributions

All authors contributed to the current study. JW and ZL conceived and designed the experiments; JW, MK, and YL performed the experiments. JW, YL, CY, YC, HH, HX, and MK helped on the data collection and analysis; JW and YL wrote the paper; MK and ZL revised the manuscript.

## Funding

This work was supported by the grants of National Natural Science Foundation of China (81703803), Guangdong Natural Science Foundation (2017A030310464), Project of Guangzhou University of Chinese Medicine (QNYC20190103), Guangdong Key Laboratory for translational Cancer research of Chinese Medicine (2018B030322011), and US NIH grants to ZL (ES026200 and HL148196).

## Conflict of Interest

The authors declare that the research was conducted in the absence of any commercial or financial relationships that could be construed as a potential conflict of interest.

## **Abbreviations:**

FMNT, formononetin; HUVECs, human umbilical vein endothelial cells; eNOS, endothelial nitric oxide synthase; NO, nitric oxide; Erk1/2, extracellular regulated protein kinase 1/2; p-Erk1/2, phosphorylated Erk1/2; Akt, protein kinase B; p-Akt, phosphorylated Akt; PPI, protein–protein interaction; GO, gene ontology; KEGG, kyoto encyclopedia of genes and genomes.
