# Supplementary material for: Simultaneous Activation of Erk1/2 and Akt Signaling is Critical for Formononetin-Induced Promotion of Endothelial Function
Source: Front Pharmacol. 2021 Jan 11;11:608518. doi: 10.3389/fphar.2020.608518 (PMC7832036; doi:10.3389/fphar.2020.608518)
Supplement: Supplementary file 1 [file datasheet1.docx]

**Supplementary** **figure legends**

**Supplementary Figure 1: Pairwise correlation analysis.** (A) Heat map of the cell growth, proliferation, migration, tube formation and intracellular NO levels. The heat map was generated by using HemI 1.0.3.7 software. Pairwise correlation between FMNT-induced intracellular NO levels and cell growth (B), proliferation (C), migration (D and E), or tube formation (F), respectively (linear regression analysis, Pearson’s correlation).

**Supplementary Figure 2: FMNT induced eNOS phosphorylation in HUVECs.** HUVECs were treated with FMNT at 10, 20, and 40 μM for 24 h. (A) Protein levels of phosphorylated eNOS (p-eNOS) were evaluated with immunofluorescence assay. The scale bar represented 25 μm. (B) Pairwise correlation between FMNT-induced intracellular NO levels and eNOS phosphorylation (linear regression analysis, Pearson’s correlation). ^**^*p* < 0.01 compared with the control group.

**Supplementary Figure 3: Pairwise correlation analysis.** (A) Heat map of the cell growth, proliferation, migration, tube formation, intracellular NO levels, eNOS activation and Erk1/2 activation. The heat map was generated by using HemI 1.0.3.7 software. Pairwise correlation between FMNT-induced Erk1/2 phosphorylation and eNOS phosphorylation (B), intracellular NO levels (C), cell growth (D), proliferation (E), migration (F and G), and tube formation (H), respectively (linear regression analysis, Pearson’s correlation).

**Supplementary Figure 4: Pairwise correlation analysis.** (A) Heat map of the cell growth, proliferation, migration, tube formation, intracellular NO levels, eNOS activation and Akt activation. The heat map was generated by using HemI 1.0.3.7 software. Pairwise correlation between FMNT-induced Akt phosphorylation and eNOS phosphorylation (B), intracellular NO levels (C), cell growth (D), proliferation (E), migration (F and G), and tube formation (H), respectively (linear regression analysis, Pearson’s correlation).

**Supplementary Figure 5: FMNT-induced interaction between Erk1/2 and Akt signaling. Protein expression was detected with Western blot analysis.** (A) Erk1/2 phosphorylation was detected in HUVECs pre-exposed to siAkt transfection before incubation with FMNT (20 μM) for 24 h. (B) Akt phosphorylation was detected in HUVECs pre-exposed to siErk1/2 transfection before incubation with FMNT (20 μM) for 24 h. The data were presented as mean ± SD (n = 3). ^***^*p* < 0.001 compared with the siCon group. ^#^*p* < 0.05 compared with the siCon + FMNT-20 group. Statistical differences were verified with Student’s *t*-test by SPSS 19.0 for all groups.

**Supplementary Figure 6：Prediction analysis for the interactions between MAPK and PI3K-Akt signaling pathways on the activation of eNOS by FMNT using network pharmacology.** (A) MAPK (including 38 targets) and PI3K-Akt (including 39 targets) signaling pathways involved in the effects of FMNT against ischemia were identified using KEGG pathway enrichment analysis with 16 common targets for both MAPK and PI3K-Akt signaling pathways. (B) The protein–protein interaction network of the 16 targets were obtained from the STRING database and produced using Cytoscape 3.7.2 software, and revealed that interactions could occur only between 9 targets.

**Supplementary Figure 1**

**
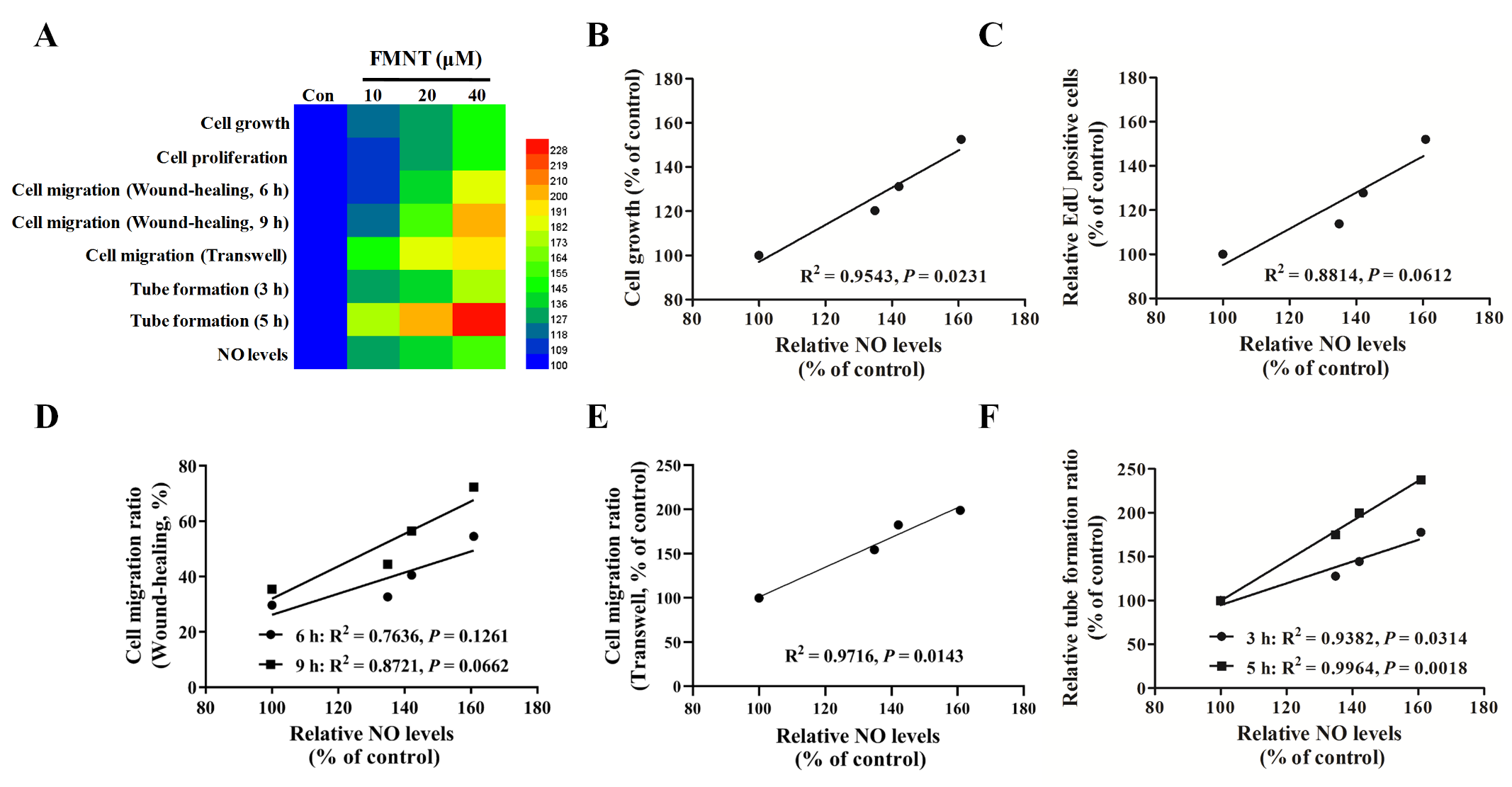
**

**Supplementary Figure 2**


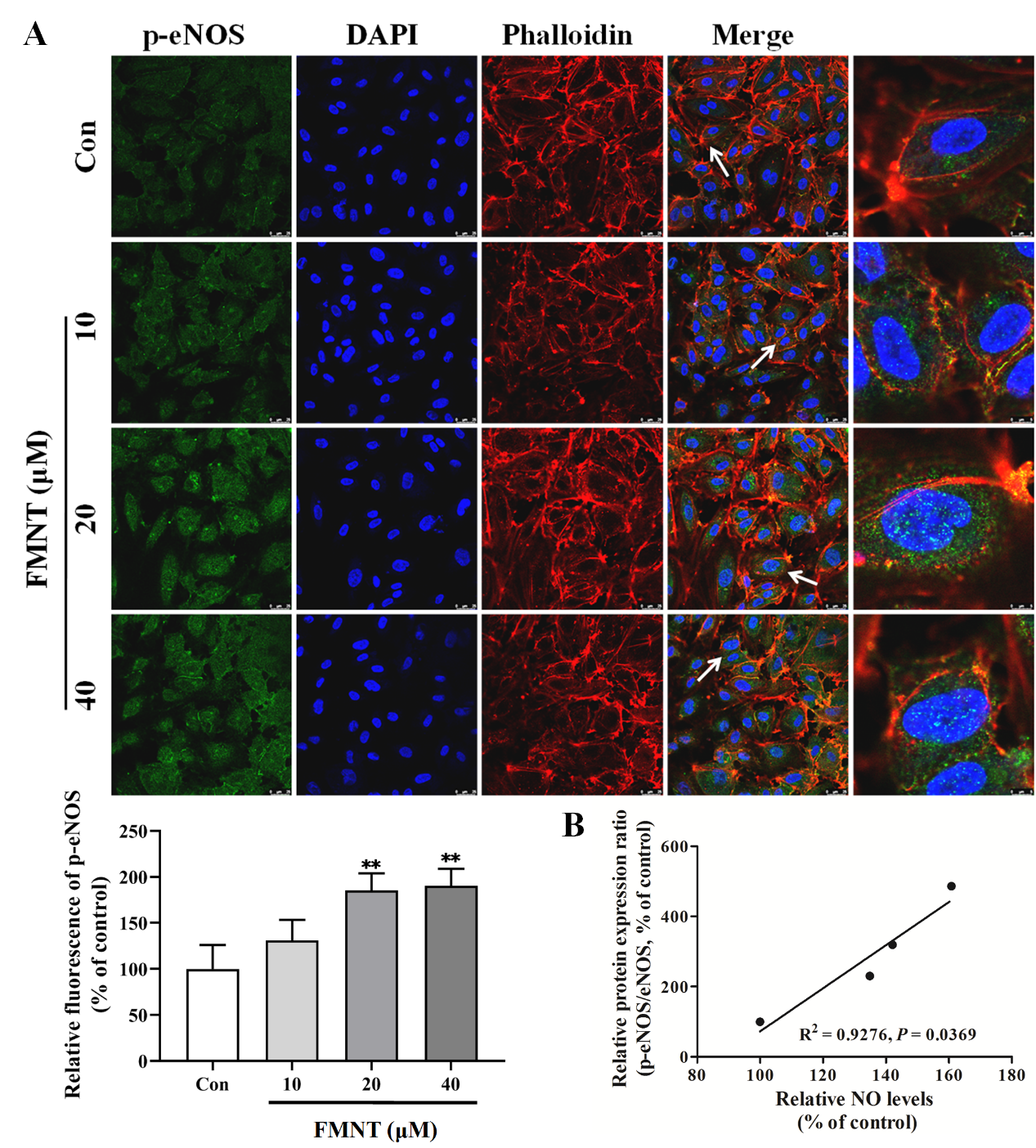


**Supplementary Figure 3**


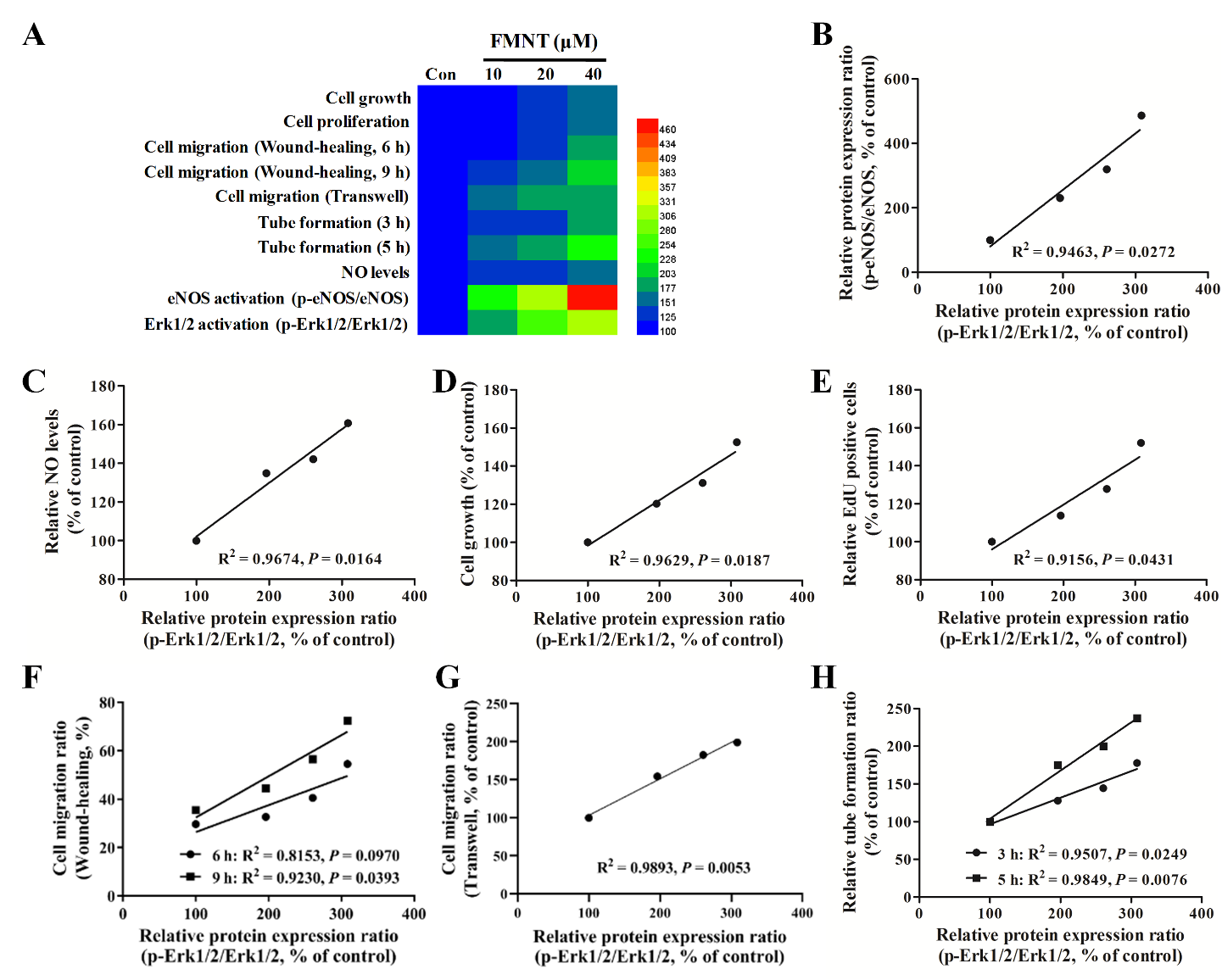


**Supplementary Figure 4**

**
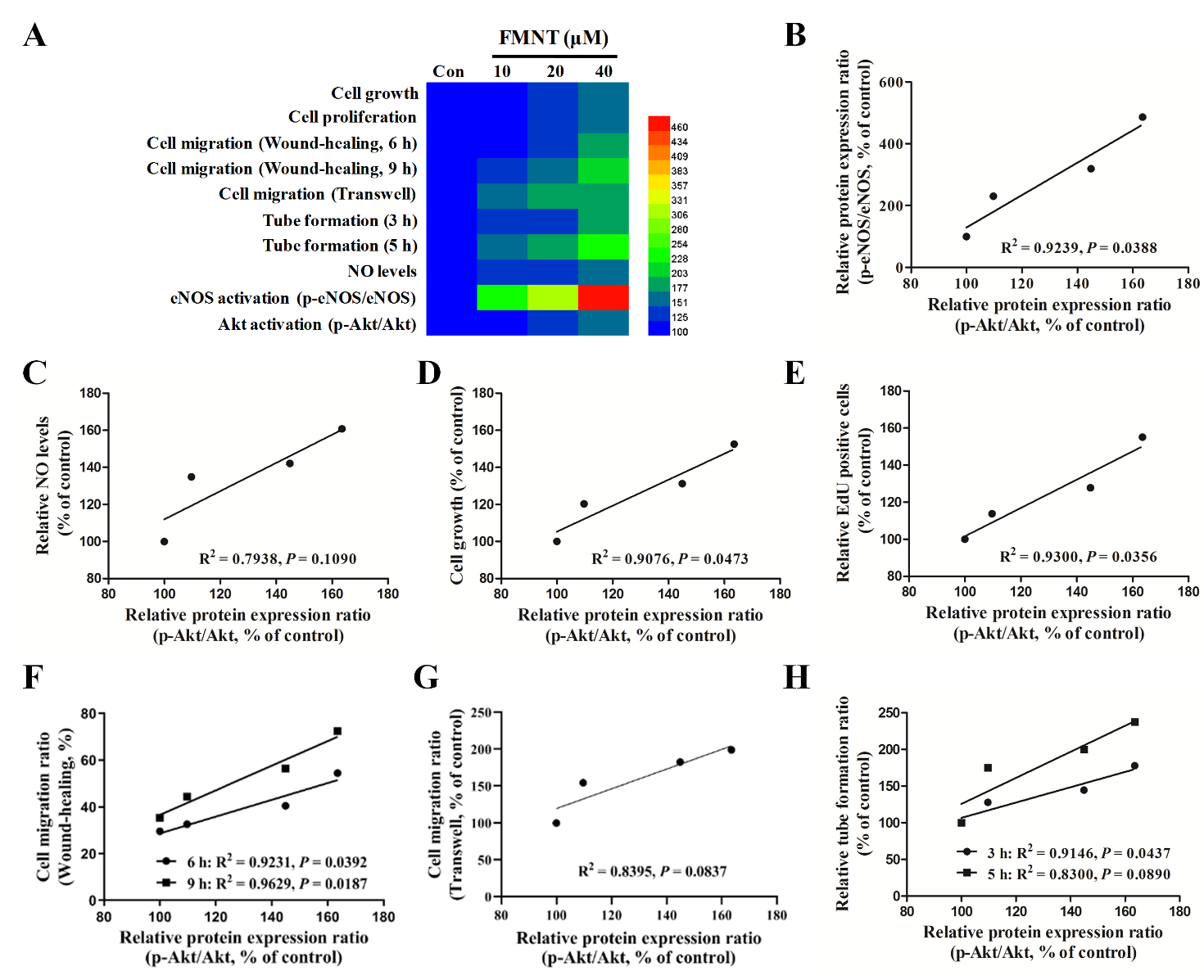
**

**Supplementary Figure 5**


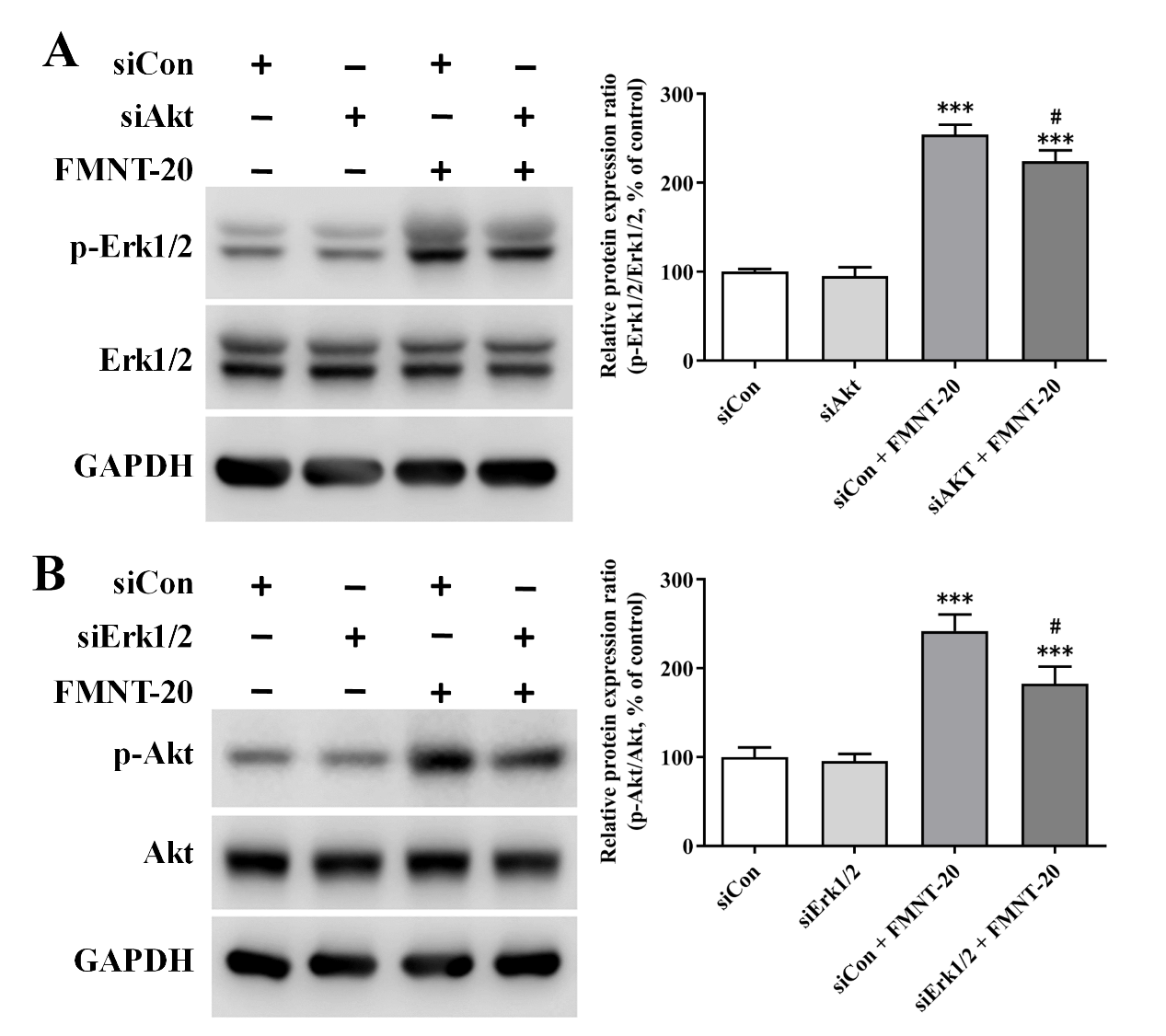


**Supplementary Figure 6**

**
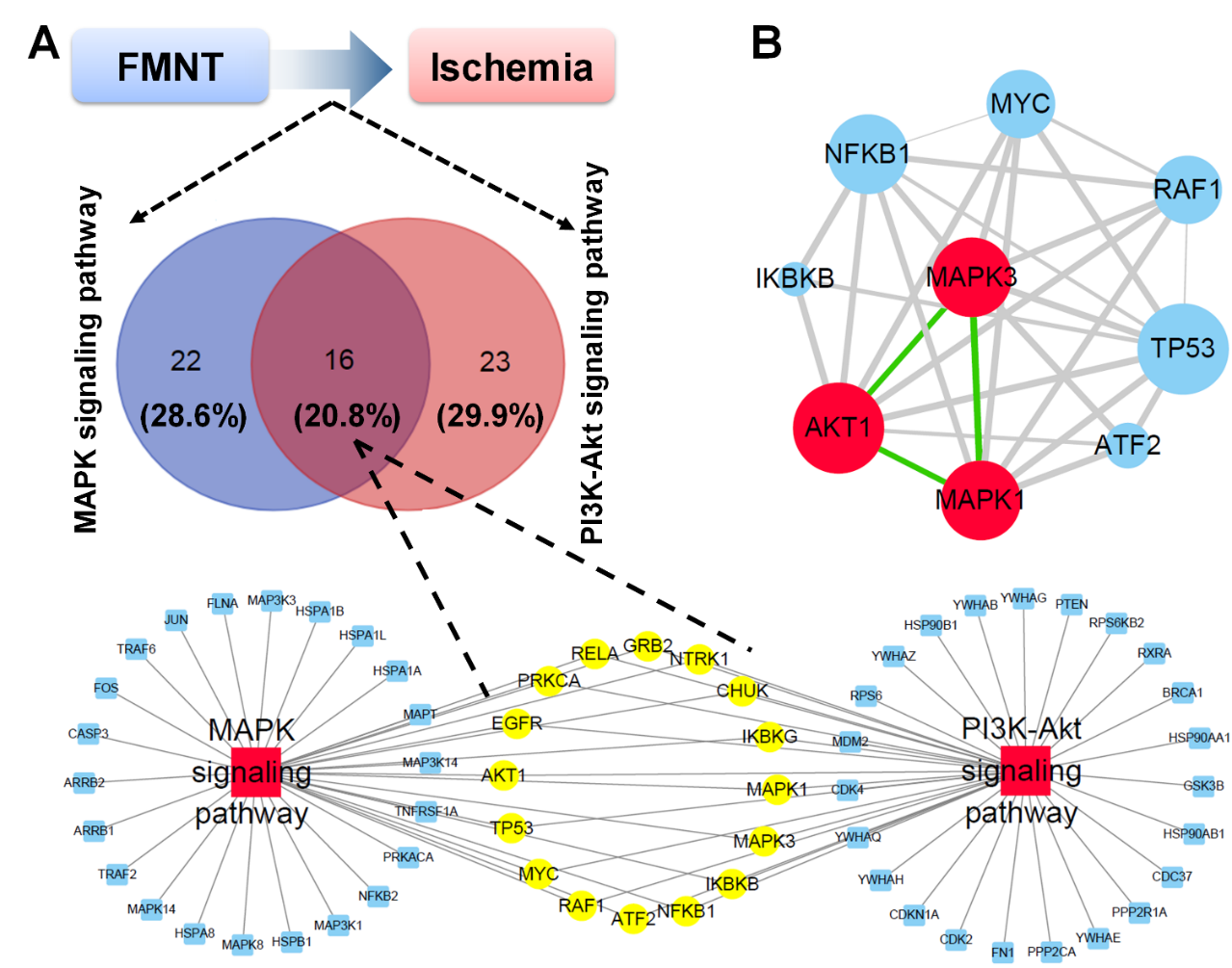
**
